# Glucose Availability Alters Gene and Protein Expression of Several Newly Classified and Putative Solute Carriers in Mice Cortex Cell Culture and *D. melanogaster*

**DOI:** 10.3389/fcell.2020.00579

**Published:** 2020-07-07

**Authors:** Mikaela M. Ceder, Emilia Lekholm, Axel Klaesson, Rekha Tripathi, Nadine Schweizer, Lydia Weldai, Sourabh Patil, Robert Fredriksson

**Affiliations:** ^1^Molecular Neuropharmacology, Department of Pharmaceutical Biosciences, Uppsala University, Uppsala, Sweden; ^2^Pharmaceutical Cell Biology, Department of Pharmaceutical Biosciences, Uppsala University, Uppsala, Sweden

**Keywords:** solute carriers, major facilitator superfamily, transporters, glucose, primary cortex cultures, deprivation, starvation, *D. melanogaster*

## Abstract

Many newly identified solute carriers (SLCs) and putative transporters have the possibility to be intricately involved in glucose metabolism. Here we show that many transporters of this type display a high degree of regulation at both mRNA and protein level following no or low glucose availability in mouse cortex cultures. We show that this is also the case in *Drosophila melanogaster* subjected to starvation or diets with different sugar content. Interestingly, re-introduction of glucose to media, or refeeding flies, normalized the gene expression of a number of the targets, indicating a fast and highly dynamic control. Our findings demonstrate high conservation of these transporters and how dependent both cell cultures and organisms are on gene and protein regulation during metabolic fluctuations. Several transporter genes were regulated simultaneously maybe to initiate alternative metabolic pathways as a response to low glucose levels, both in the cell cultures and in *D. melanogaster.* Our results display that newly identified SLCs of Major Facilitator Superfamily type, as well as the putative transporters included in our study, are regulated by glucose availability and could be involved in several cellular aspects dependent of glucose and/or its metabolites. Recently, a correlation between dysregulation of glucose in the central nervous system and numerous diseases such as obesity, type 2 diabetes mellitus as well as neurological disease such as Alzheimer’s and Parkinson’s diseases indicate a complex regulation and fine tuning of glucose levels in the brain. The fact that almost one third of transporters and transporter-related proteins remain orphans with unknown or contradictive substrate profile, location and function, pinpoint the need for further research about them to fully understand their mechanistic role and their impact on cellular metabolism.

## Introduction

The central nervous system (CNS) requires large amounts of energy compared to its size to maintain its signaling activities, however, both too much and too little energy can have serious implications for brain activity and the body (Ames, 2000; Bruce-Keller et al., 2009). Glucose is the main energy source for the brain and, while it can be supplemented, it cannot be replaced. Glucose is the source of ATP needed for activity, through its intermediary form glucose-6-phosphate (Glc-6-P). Glc-6-P is also the substrate for the pentose phosphate shunt pathway (PPP) to generate NADPH, vital in managing oxidative stress and to synthesize nucleic acid precursors (Mergenthaler et al., 2013). In addition, glucose is pivotal for synthesizing compounds that are not available due to the blood brain barrier (BBB), such as glutamate, aspartate, glycine, D-serine, glycoproteins, glycolipids, and neurotransmitter precursors (Dienel, 2012). Understanding all the components involved in keeping a correct glucose concentration in cells of the nervous system is of great value and numerous diseases are coupled to aberrant glucose regulation. More than 10% of early-onset absence epilepsy is caused by mutations in the *SLC2A1* gene encoding GLUT1, a glucose transporter (Arsov et al., 2012). A reduction of glucose metabolism is one of the earliest signs of Alzheimer’s disease (AD), and a disturbed glucose metabolism is associated with progression of the disease (Hoffmann et al., 2013). In addition, metabolic disorders such as obesity and type 2 diabetes mellitus are also linked to both AD progression and cognitive impairment (Kapogiannis and Mattson, 2011).

The solute carriers (SLCs) is an important family of proteins capable of transporting vast number of molecules, including glucose, neurotransmitters, and drugs across membranes. While many are characterized, several have unknown substrate profiles and expression (Cesar-Razquin et al., 2015; Lin et al., 2015). One well characterized SLC family is the glucose transporters of the SLC2 family, GLUT1–14, which belongs to the Pfam classification Major Facilitator Superfamily (MFS; Perland and Fredriksson, 2017). The 14 members have different expression patterns (Thorens and Mueckler, 2010), are tightly controlled to provide the optimal intake of glucose in cells (Fladeby et al., 2003), and can operate under different concentration gradients. GLUT1 facilitates glucose transport through the BBB and into astrocytes, oligodendrocytes and microglia where the glucose concentrations are steep, while GLUT3, with a higher transport rate, facilitates glucose uptake into neurons (Simpson et al., 2007; Thorens and Mueckler, 2010). In additions, different metabolic states such as synaptic activity in neurons increase surface expression of GLUT3 (Ferreira et al., 2011). Many transporter proteins are highly conserved and while the exact distribution and localization might not be the same across species, the function is more often conserved. Phylogenetic analysis of transporters belonging to the MFS result in function based clustering rather than lineage based (Vishwakarma et al., 2018). GLUT1 is also responsible for glucose uptake in *Drosophila melanogaster*, however, not across the BBB as in humans, but rather into neurons in the brain (Volkenhoff et al., 2018).

Perland and Fredriksson (2017) identified proteins classified as MFS that could be putative SLCs, proteins similar to SLCs which were not currently well characterized or classified into any of the existing SLC families. Recently, MFSD2A, MFSD2B, MFSD3, MFSD4A, MFSD4B, MFSD5, MFSD10, SPNS1, SPNS2, SPNS3, SV2A, SV2B, SV2C, SVOP, and SVOPL were categorized into existing or new SLC families and obtained a new gene name according to the SLC nomenclature (Yates et al., 2017). Some of these newly classified SLCs and putative SLCs (from now on called new SLCs and putative transporters) do seem to have a direct or indirect involvement in nutrient metabolism. SV2A (SLC22B1) might function as a galactose transporter (Madeo et al., 2014), MFSD2A, recently included into the SLC59 family, transports fatty acids such as docosahexaenoic acid (DHA; Nguyen et al., 2014) and is important in the formations of the brain and BBB (Ben-Zvi et al., 2014), MFSD4B, now called *Slc60a2*, transports glucose and fructose (Horiba et al., 2003a, b). In addition, *Mfsd1* and *Mfsd3* (presently called *Slc33a2*) (Perland et al., 2017b), *Mfsd5* (now under the name of *Slc61a1)* and *Mfsd11* (Perland et al., 2016), *Mfsd14a* and *Mfsd14b* (Lekholm et al., 2017), and *Unc93a* (Ceder et al., 2017) are all affected by energy availability mice *in vivo* and *Mfsd9* is linked to diabetes (Rampersaud et al., 2007). Amino acid starvation affects both *Mfsd2a* and *Mfsd11* (Hellsten et al., 2017) in mouse hypothalamic cell line N25/2.

In order to shed more light on these SLCs of MFS type, we focused on their involvement in metabolism and their involvement in glucose response. Phylogenetical analysis revealed conservation between the repertoire of these putative SLCs in man and mice, and for most cases also in *D. melanogaster*. The promotor regions of these putative SLCs indicate that their expression could be regulated by nutritional signals, as a majority contained regulatory motifs linked to nutrition. In addition, we studied changes in gene expression in both mouse embryonic cortex cultures and *D. melanogaster* subjected to D-glucose starvation and saw that many of the putative transporters were indeed affected by lack of nutrients. Furthermore, as SLCs have been found to be regulated by changes in intracellular localization and expression levels, protein localization in cortex cultures was followed for a few of the putative SLCs to monitor their shift in localization during glucose starvation and also here, changes in both expression level and localization in response to nutritional status could be observed. Refeeding glucose to starved cortex cultures did return some of the gene expression changes back to normal as did the feeding of starved adult male flies, indicating a dynamic and fast regulation of some of these new transporters. Low glucose levels in primary cortex cultures resulted in an even greater change in expression levels than starvation did, and similarly, a low sugar diet in flies increased the expression of several orthologues to the putative transporters in this study. While their exact function is still not known, many of the putative transporters studied here are highly dynamic in their expression, affected by glucose starvation and deprivation. They have a high probability of being necessary for, not just normal neuronal function, but also during fluctuating energy and glucose availability.

## Materials and Methods

### Phylogenetic Analysis

The phylogenetic relationship between the human SLC22A32, SLC22B1, SLC22B2, SLC22B3, SLC22B4, SLC22B5, SLC33A2, SLC49A3, SLC59A1, SLC59A2, SLC60A1, SLC60A2, SLC61A1, SLC63A1, SLC63A2, SLC63A3, MFSD1, MFSD6, MFSD6L, MFSD7B, MFSD7C, MFSD8, MFSD9, MFSD11, MFSD12, MFSD13A, MFSD14A, MFSD14B, UNC93A, and UNC93B1 and their relative protein sequences in *Mus musculus* and *D. melanogaster* were investigated, Supplementary Data 1. The human and mouse protein sequences were downloaded from UniProt (2019). The human protein sequences were then used to build a Hidden Markov model (HMM) using HMM_BUILD_ from the HMMER package (Eddy, 2011). The HMM was used to search for relative sequences in the *D. melanogaster* proteome (BDGP6.pep.all). MAFFT (Katoh et al., 2019) was used to align the sequences and mrBayes 3.2.2 (Huelsenbeck et al., 2001) software was used to generate the tree, and the procedure was run on a non-heated chain with two runs in parallel (*n* run = 2) under the mixed amino acid model with eight gamma categories and invgamma as gamma rates for a total of 2,000,000 generations.

### Promoter Sequences Analysis

The presence of promoter sequences and transcription factor motifs for the putative SLCs in mouse were investigated using the Eukaryotic Promoter Database (Cavin Perier et al., 1998). The gene sequences, 1000 basepair upstream to 100 basepair downstream of the transcription start site were used for each putative transporter and the SLCs includeded in the study. The promoter Motifs library was used to search for promoter sequneces. Three promoter motifs, the TATA box, CCAAT box and the GC box were analyzed. The JASPER CORE 2018 vertebrate library was used to search for transcription factor motifs and several transcriptions factors known to be affected by macronutirent metabolism were analyzed; *Atf4* (Averous et al., 2004; Dey et al., 2012), *Cebpa* (Pedersen et al., 2007), *Cebpb* (Ramji and Foka, 2002), *Creb1* (Kim et al., 2016; Steven et al., 2017), *E2f1* (Denechaud et al., 2017), *FoxO1* (Kousteni, 2012), *Mlx*, *Mlxip*, and *Mlxipl* (Ma et al., 2006; Havula and Hietakangas, 2012).

### Animals

All procedures involving mice were approved by the local ethical committee in Uppsala (Uppsala Djurförsöksetiska Nämnd, Uppsala District Court, permit number C39/16, C67/13, and C419/12) in unity with the guidelines of European Communities Council Directive (2010/63). All animals were maintained in a temperature-controlled room on a 12 h light:dark cycle where they had free access to water and food. C57Bl6/J (Taconic M&B, Denmark) females and males were used.

### Cell Culture

Wildtype mice were mated and at e15 the females were euthanized and the embryos were removed. The females were not further used. The embryos were decapitated and cortex tissue was dissected and used for primary cultures. Culture set-up is previously described in Perland et al. (2016).Briefly, the cortex samples were pooled, washes in PBS-Glucose and dissociated using papain (Thermo Fisher Scientific) and DNAse (Thermo Fisher Scientific) for 30 min. Thereafter, mechanical dissociation was performed by pipetting before filtering the cell solution through a cell strainer (Thermo Fisher Scientific). The cells were diluted in plating media consisting of DMEM:F12 (Gibco, Invitrogen) supplemented with 2 mM GlutaMax, 1 mM Na-Pyruvate, 10% FBS and 1% Pen strep, all supplied from Invitrogen, and plated on Poly-L-Lysine (Sigma) coated coverslips (12 mm, #1.5) (Menzel-Gläser, Thermo Fischer Scientific) in 24-well plates (Nonclone delta, Thermo Fischer Scientific) or on Poly-L-Lysine coated 6-well plates (Nunclone delta, Thermo Fischer Scientific). The cell was incubated at 37°C, 5% CO_2_ for 3 h before media change to NeurobasalA media (Gibco, Invitrogen) supplemented with 2 mM Glutamax, 1 mM Na-Pyruvate, 1 % Pen-strep and 1x B27 Supplement. 75% of the media was changed every third day and the cells allowed to grow for 9 days.

#### Glucose Starvation and Starvation and Refeeding and Deprivation in Primary Cortex Cells

To investigate gene and protien expression changes compared with the general culturing conditions of primary cortex cultures, glucose starvation and derpivation were performed, as well as glucose starvation followed by refeeding. Glucose starvation of the cell cultures was performed by changing media to NeurobasalA with 0 g/L D-Glucose (A2477501, Thermo Fisher Scientific), 2 mM Glutamax, HEPES, 1% Pen-strep and 1x B27 without insulin (A1895601, Thermo Fisher Scientific). Starvation was performed for 3 and 12 h. The starved and refed group was subjected to the same condition as the starved group for 3 and 12 h, before refeeding for 12 h on regular NeurobasalA media containing 4.5 g/L D-Glucose, 2 mM Glutamax, Hepes, 1% Pen-strep and 1x B27. The control groups were kept on NeurobasalA media, but underwent equal number of media change as the corresponding experimental group.

Sugar deprivation of the cell culture was performed by changing the media to DMEM containing 1 g/L D-Glucose (11054020, Thermo Fisher Scientific), 2 mM Glutamax, HEPES, 1% Pen-strep, and 1x B27. The deprivation was performed for 3 and 12 h. The control group was kept on DMEM containing 4.5 g/L D-Glucose (same concentration as in NeurobasalA Media), 2 mM GlutaMax, HEPES, 1% Pen-strep and 1x B27 Supplement (all from Invitrogen) and underwent equal number of media changes as the deprived cells.

### Fly Stocks and Maintenance

To study how starvation and sugar alters the gene expression of the putative SLCs in *D. melanogaster*, CSORC flies (cross between CantonS and OregonR-C, kindly donated by Michael J. Williams, Uppsala University) were used. The stock was maintained at 25°C, humidity 50%, on a 12 h light:dark cycle and with free access to enriched Jazz mix standard food (Thermo Fisher Scientific, Sweden). Virgin male flies were collected and raised to five days old before experiments were performed. Five to eight replicates were used, with ten flies in each replicate, depending on the experimental setup, see details below.

### Sugar Starvation in Adult Male Flies

Flies were subjected to 0 h (control, *n* = 6), 3 h (*n* = 8), 12 h (*n* = 6) of starvation, as well as 3 h (*n* = 8) and 12 h (*n* = 6) of starvation followed by refeeding. Five days old male flies were transferred to vials prepared with 1% agarose for each group. The control group was regularly fed male CSORC flies. The flies subjected to starvation and refeeding were transferred to vials with 8 ml regular food after starvation for 12 h. Whole flies were euthanized by freezing in −80°C followed by RNA extraction.

### Sugar Diets in Adult Male Flies

Adult male flies (*n* = 5) were starved for 24 h before kept on varying concentrations (g/dl) of sucrose (Sigma Aldrich) and yeast extract (VWR) in 1% agarose for 5 days. Four concentrations were used 10 g/dl sugar and yeast (control), 2.5 g/dl sugar and yeast (low calorie diet), 2.5 g/dl sugar and 10 g/dl yeast (low sugar diet) and 40 g/dl sugar and 10 g/dl yeast (enriched sugar diet). Whole flies were euthanized by freezing in −80°C followed by RNA extraction.

### RNA Preparation and cDNA Synthesis

*Primary cortex cultures*: six wells per group (*n* = 6) were used for RNA extraction, except for the starved and refed control group where five wells were used. The RNA was retrieved using Allprep DNA/RNA micro kit (Qiagen), according to the manufacturer’s instructions. Concentrations were measured using ND-1000 spectrophotometer (NanoDrop Technologies). Two microgram RNA template was used for the cDNA synthesis, performed according to manufacturer recommendations using the Applied Biosystems High Capacity RNA-to-cDNA kit (Invitrogen). cDNA concentration was measured using a ND-1000 spectrophotometer and diluted to 20 ng/μl with sterile water.

*Drosophila melanogaster*: Total RNA and cDNA were acquired as described in Chomczynski and Sacchi (1987), Williams et al. (2016). *RNA extraction*: Briefly, flies were homogenized in 60 μl Trizol (Invitrogen). 650 μl Trizol was added followed by incubation for five minutes at room temperature. Hundred and sixty microliter of chloroform (Sigma Aldrich) was added, samples were shaken and incubated for 4 min followed by centrifugation at 14,000 rpm (microcentrifuge from Thermo Fisher Scientific, 24 × 1.5/2.0 ml rotor) at 4°C for 12 min. The upper phase was transferred to a new Eppendorf tube, precipitated with 400 μl isopropanol (Sigma Aldrich) before stored at −20°C for 30 min before centrifugation. The supernatant was discarded, and the RNA pellet was washed three times in 75% ethanol (Sigma Aldrich) and spun for 5 min. During the second wash, DNase treatment (Thermo Fisher Scientific, DNase I, RNase Free, 1 U/μl) was added. Pellet air-dried for 15 min, before dissolved in 20 μl of RNAse-free water. Concentration was measure using a ND-1000 spectrophotometer (NanoDrop Technologies). *cDNA synthesis*: 2 μg RNA template was used; cDNA was synthesized with High Capacity RNA-to-cDNA kit (Applied Biosystems) according to manufacturer’s instructions. The samples were diluted to 10 ng/μl with sterile water.

### Primer Design and Quantitative Real-Time PCR

Gene expression and expression changes were determined using quantitative real-time PCR (qRT-PCR). All primers were designed using Beacon Design 8 (Premier Biosoft) (Table 1).

**TABLE 1:** Primers for qRT-PCR. Name of the primers designed for mouse primary cortex cultures are marked with mm and dm for *D. melanogaster.*

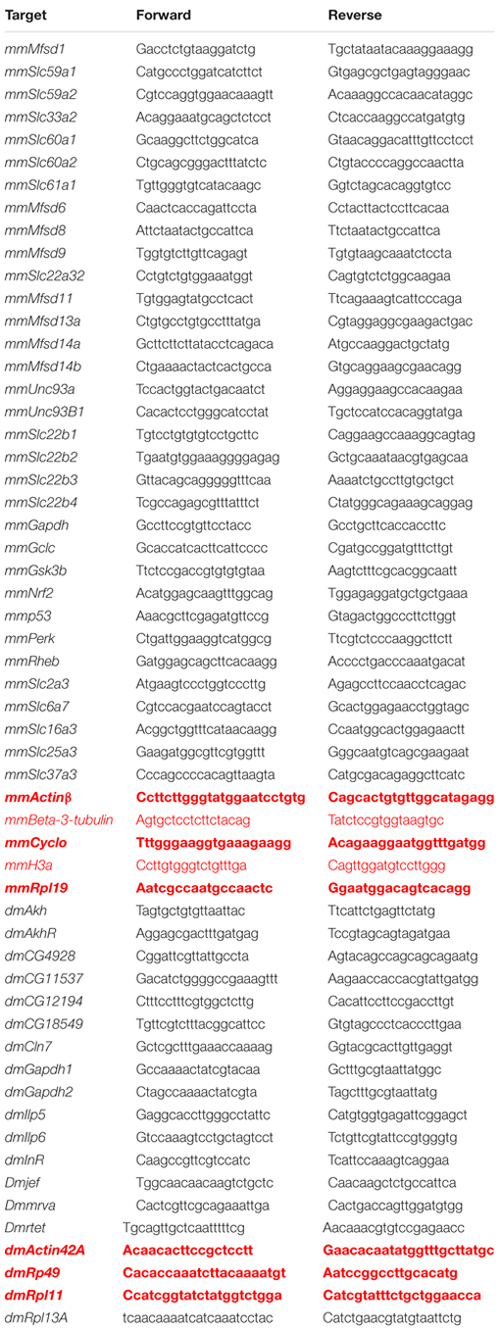

*Housekeeping genes are marked in red and they housekeeping genes that were used for normalization in bold.*

Final volume for each qRT-PCR reaction was 10 μl (for qPCR using fly samples) or 20 μl (for qPCR using cell samples) consisting of: 3 μl cDNA (20 ng/μl) for primary cortex cultures and 2.5 μl cDNA (10 ng/ul) for flies, 0.05 μl of each primer (100 pmol/μl), 3.6 μl 10× DreamTaq buffer (Thermo Fischer Scientific), 0.2 μl of 25 mM dNTP mix (Thermo Fisher Scientific), 1 μl DMSO, 0.5 μl SYBR Green (Invitrogen) and 0.08 μl of Dream Taq (5 U/μl, Thermo Fisher Scientific). The volume was adjusted to final volume with sterile water. An iCycler real-time detection instrument (Bio-Rad) was used with the following settings: initial denaturation for 30 s at 95°C, 55 cycles of 10 s at 95°C, 30 s at 55–61°C (optimal temperature depending on primer) and 30 s at 72°C. A melting curve was generated by heating from 55 to 95°C with 0.5°C increment at 10 s dwell time and a plate read at each temperature. All qRT-PCR were run in triplicates and a negative control was included on each plate. All data was collected using the MyIQ (Bio-Rad Laboratories) software.

### Analysis of qRT-PCR Data

Primer efficiency was calculated with LinRegPCR software, followed by Grubbs test (GraphPad software) to remove outliers before calculations were corrected for each primer based on primer efficiency. The GeNorm protocol (Vandesompele et al., 2002) was used to find stable housekeeping genes, see Table 1 for details about primers. The expression was then normalized using Geomean values from the stable housekeeping genes. In total three housekeeping genes were used and the same, species-specific housekeeping genes were used for all data sets (Mouse: *Actinβ*, *Cyclo* and *Rpl19*, *D. melanogaster: Actin42A*, *Rp49* and *Rpl11*). The primer corrected and normalized mean (±SD for primary cortex cultures, ±SEM for flies) for each gene were calculated and plotted; heat-maps control set to 1, graphs control set to 100%. Outliers were removed using the Grubbs outlier test with α = 0.05 before proceeding with statistics. For primary cortex cultures, Mann–Whitney were performed for gene analysis of expression changes, where ^∗^*p* < 0.05, ^∗∗^*p* < 0.01, ^∗∗∗^*p* < 0.001. For flies differences were calculated using Kruskal-Wallis test were performed and Mann–Whitney with Bonferroni’s multiple correction (adjusted *p*-values^∗^*p* < 0.0489, ^∗∗^*p* < 0.00995, ^∗∗∗^*p* < 0.00099). If only two biological replicates or less (e.g., *Slc60a2* and *Nrf2*) provided a RFU (relative fluorescence unit) value, no statistic could be performed.

GENESIS version 1.7.6 was used to create heat-maps over the gene expression alterations as described in Hellsten et al. (2017). Briefly, the fold change was calculated between the control and the experimental groups. All genes were kept in the heat-map. Red gradient indicates the level of upregulation, blue gradient indicates the level of downregulation, while gray boxes represent missing values.

### Fluorescent Immunocytochemistry

At the termination of the experiment, primary cortex cells for ICC were washed once with DPBS (Gibco, Invitrogen) and fixed using 4% Formaldehyde (Histolab) for 60 min. The cells were then washed 3 × 10 min with DPBS and stored in DPBS at 4°C until analysis. Immunocytochemistry (ICC) was performed with a set of antibodies against putative SLCs that have been verified elsewhere and that provided similar immunostaining patterns between the published ICCs (Perland et al., 2017c) and the ICCs performed in this study. Immunocytochemistry was performed as described in Lekholm et al. (2017) with antibodies for MFSD1 [SAB3500575, Sigma, (Perland et al., 2017b)] diluted 1:50 in Supermix blocking solution, SLC60A1 [SAB1305276, Sigma, (Perland et al., 2017c)] diluted 1:100, MFSD11 [Sc-243473, Santa-Cruz, (Perland et al., 2016)] diluted 1:80, MFSD14A [Ab103836, Abcam, (Lekholm et al., 2017)] diluted 1:100, MFSD14B [SAB2107506, Sigma, (Lekholm et al., 2017)] diluted 1:100, UNC93A [Ab69443, Abcam, (Ceder et al., 2017)] diluted 1:100 also in Supermix together with PAN diluted 1:200 (Millipore), GFAP diluted 1:200 (Abcam) and DAPI (Sigma Aldrich) diluted in PBS 1:15,000. For fluorescent quantification, images were taken using the same exposure settings for all pictures using an Olympus microscope BX53 with an Olympus DP73 camera and CellSens Dimension software. For each group, 6–10 pictures were taken with several cells in each picture. The images were processed in CellProfiler version 2.2.0. For details about pipline and images used, see Supplementary Data 2 and Figures 5, 6, 9. Identification of cellular compartments, nuclei (based on DAPI staining) and whole neuronal cytoplasm (based on PAN-neuronal staining) were performed. The mean pixel intensity in protein staining was measured in each subcellular compartment and the average fluorescent intensity per cell was calculated. Graphpad Prism version5 was used to generate graphs, mean (±SEM), and differences were calculated using unpaired *t*-test (^∗^ > 0.05, ^∗∗^ > 0.01, ^∗∗∗^ > 0.001).

### Western Blot

Western blot analysis was performed as previously described in Lekholm et al. (2017); Hellsten et al. (2018). Briefly, cells were washed in PBS and lysed in 1000 μl of lysis buffer (150 mM NaCl, 50 mM Tris, 4 mM KCl, 1 mM MgCl2, 1 mM Na_3_VO_4_, 10% glycerol, 1% Nondiet P-40, and protease inhibitors). Total protein was quantified using the Bradford method (Bio-Rad), and equal concentrations of protein samples were prepared using 10 μl (∼9–11 μg) of protein samples were diluted in 15 μl of sample buffer [95% 2 × Lammeli’s sample buffer (Bio-Rad), 5% 2-mercaptoethanol (Sigma Aldrich)]. and separated on 10% Mini-PROTEAN^®^ TGX^TM^ Precast Protein Gels (Bio-Rad). Proteins were transferred to a 0.2 μm PVDF membrane using the Trans-Blot^®^ Turbo^TM^ Mini PVDF Transfer Packs (Bio-Rad) in the Trans-Blot^®^ Turbo^TM^ Transfer System for 10 min (Bio-Rad). Blots were blocked in BSA (5% w/v in PBS +0.1 % Tween 20) for 1 h at room temperature. The following primary antibodies were used according to the manufacturer’s instructions: mTOR (7C10 Rabbit mAb #2983, Cell Signaling) and β-actin (Sigma Aldrich) diluted 1:1000 in blocking buffer overnight at 4°C. The membrane was washed 3 × 10 min in TTBS before A HRP goat α-rabbit coupled secondary antibodies were used at 1:5000 (Cell signaling) and HRP goat α-mouse, diluted 1:10,000 (Invitrogen), for 1 h at room temperature. The membrane was developed using Clarity Western ECL Substrate (Bio-Rad) and visualized using a CCD camera (ChemiDoc, Bio-Rad) with ImageLab software for documentation. The western blots were quantified using ImageJ, Fiji edition (Schindelin et al., 2012) and the protein expression was normalized against β-actin. GraphPad Prism 5 (Graph Pad software) was used to generate graphs and for statistical calculations. Mann-Whitney was performed with significance levels (^∗^ ≤ 0.05, ^∗∗^ ≤ 0.01, ^∗∗∗^ ≤ 0.001).

## Results

**FIGURE 1:**
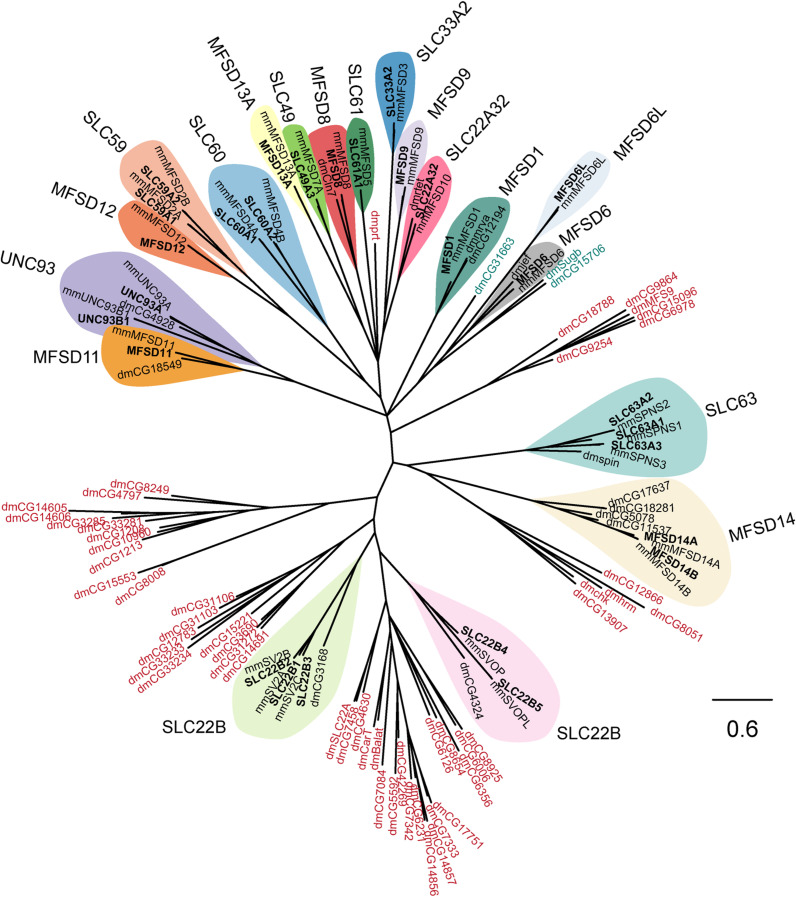
Several human putative SLCs are conserved in *M. musculus* and *D. melanogaster*. The phylogenetic relationship for putative SLCs in humans (bold text), mouse (mm), and fruit flies (dm) were investigated. The human and mouse protein sequences were downloaded from uniport.org (UniProt, 2019), according to earlier publications (de la Cruz et al., 2003). The human protein sequences were then used to build a Hidden Markov Model (HMM; Duprat et al., 2007; Eddy, 2011) that was used to search for relative proteins in the fruit fly proteome. The sequences were then combined in a multiple protein alignment using MAFFT (Katoh et al., 2019) and mrBayes (Huelsenbeck et al., 2001) was used to generate the tree, with a branch length of 0.6. All human putative SLCs had an orthologue in mouse, while orthologous proteins in fruit flies were only identified for SLC22A32 (rtet), SLC22B1–3 (CG3168), SLC22B4–5 (CG4324), SLC63A1–3 (spin), MFSD1 (mrva, CG12194), MFSD6 (jef), MFSD8 (Cln7), MFSD11 (CG18549), MFSD14A–B (CG5078, CG11537, CG17637, CG18281), and UNC93A–B1 (CG4928). The HMM also identified other proteins in fruit fly that resembles the conserved motif (MFS motif) for the putative SLCs, however, none of these proteins clustered clearly with a human putative SLC. Protein sequences in blue are proteins that clustered close with the MFSD6 and MFSD6L, while protein sequences in red are proteins belonging to other SLC families of MFS type.

Human transporters were phylogenetically analyzed regarding sequence relationships between them and their orthologues in *M. musculus* and *D. melanogaster*, Figure 1. Recently, fifteen of the putative “atypical” SLCs of MFS type, *Mfsd2a* (*Slc59a1*), *Mfs2b* (*Slc59a2*), *Mfsd3* (*Slc33a2*), *Mfsd4a* (*Slc60a1*), *Mfsd4b* (*Slc60a2*), *Mfsd5* (*Slc61a1*), *Mfsd10* (*Slc22a32*), *Sv2a* (*Slc22b1*), *Sv2b* (*Slc22b2*), *Sv2c* (*Slc22b3*), *Svop* (*Slc22b4*), *Svopl* (*Slc22b5*), *Spns1* (*Slc63a1*), *Spns2* (*Slc63a2*), and *Spns3* (*Slc63a3*) described in Perland and Fredriksson (2017); Perland et al. (2017a) were classified into different SLCs families^1^. Still, there are uncertainties about their function, expression and cellular role, hence, they were analyzed together with the remaining putative SLCs not yet placed in families. Furthermore, *Slc49a3* (*Mfsd7a*) was included due to its similar properties to the rest of the Major facilitator superfamily domain containing proteins (MFSD).

All human sequences had one orthologue in mice, while in flies, orthologues were not identified for SLC33A2, SLC59, SLC60, SLC61, MFSD9, MFSD12, and MFSD13A. Furthermore, three protein sequences in flies (CG31663, CG15706, and Sugb in green shade) were identified to share a common ancestor to the human MFSD6 and MFSD6L. In addition, six clusters of protein sequences (protein name in red shade) for *D. melanogaster* were identified by the Hidden Markov Model but none of them clustered together with a human sequence.

**FIGURE 2:**
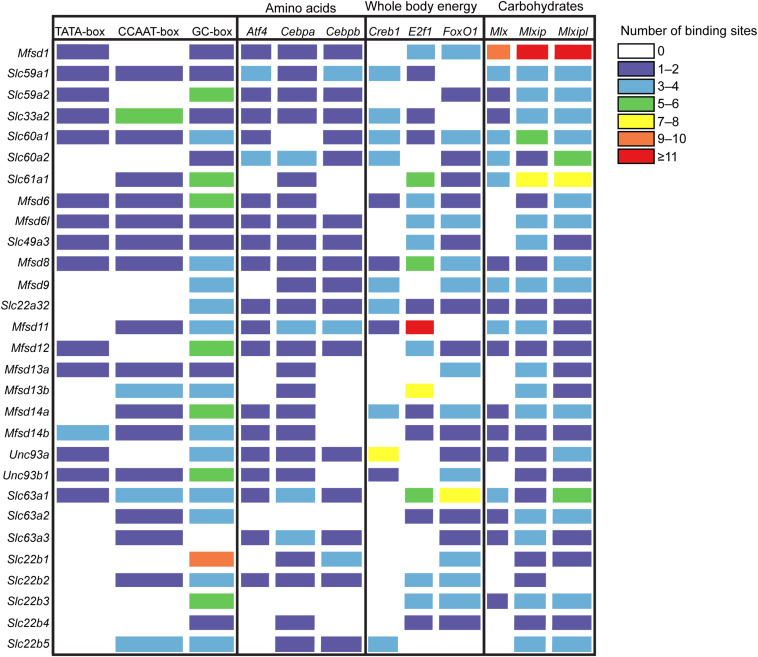
Promoter analysis of the putative SLCs using protein sequences from mouse. The gene sequence, 1000 base-pair upstream to 100 base-pair downstream of the transcription start site, for each SLCs and putative transporter were investigated, and data was collected from the Eukaryotic Promoter Database (EPD) (Cavin Perier et al., 1998). The promoter Motifs library was used to search for promoter sequences, while the JASPER CORE 2018 vertebrate library was used to search for transcription factor motifs. The transcription factor (TF) motifs investigated were *Atf4*, *Cebpa* and *Cebpb*, common amino acid sensing elements, *Creb1*, *E2f1* and *FoxO1*, TFs known to react to and regulate genes involved in energy expenditure, and *Mlx*, *Mlxip* and *Mlxipl*, carbohydrate sensing elements. The color indicates the number of binding sites present for each motif for each putative SLCs; purple 1–2, blue 3–4, green 5–6, yellow 7–8, orange 9–10, red ≥11.

Since glucose has a central role in cell physiology, cells have evolved systems to regulate genes in response to glucose availability (Towle, 2005). The promoter region of the newly identified SLCs and putative transporters and 1000 base-pair (bp) upstream of the transcriptional start site (TSS) were analyzed for predicted regulatory motifs, Figure 2, as described in Ceder et al. (2017). Of the putative SLCs of MFS type 52% had one to four TATA-boxes, 65.5% had one to six CCAAT-boxes and in all sequences at least one GC-box was predicted, except for *Slc63a3*. There are several transcription factors known to be affected by macronutrient availability in the body. *Atf4* (Averous et al., 2004; Dey et al., 2012) binding sites was present within the 1000 bp upstream of TSS for 69% of the sequences, while a majority had at least one copy of *Cebpa* or *Cebpb* (Ramji and Foka, 2002; Pedersen et al., 2007; Dey et al., 2012) binding sites. Binding sites for the cAMP-responsive element binding protein (*Creb1*) (Kim et al., 2016; Steven et al., 2017) was found in one or two copies in *Mfsd6*, *Mfsd8*, *Mfsd11*, and *Unc93b1*, three to four copies in *Slc22a32*, *Slc22b5*, *Slc33a2*, *Slc59a1*, *Slc60a1*, *Slc60a2*, *Mfsd9*, and *Mfsd14a*, seven to eight copies in *Unc93a*. The novel regulator of metabolism, *E2f1* (Denechaud et al., 2017), had predicted binding sites in 20 out of 29 transporters and 60% of them had more than three copies present, with the highest amount of copies in *Mfsd11*. *FoxO1* (Gross et al., 2008; Kousteni, 2012) binding sites was predicted to be present in all sequences except five; *Slc22b5*, *Slc33a2*, *Slc59a1*, *Mfsd11*, and *Mfsd13b*. A majority (96.5%) of the sequences harbored at least one copy of the carbohydrate-responsive element binding protein (*Mlxipl*) (Ma et al., 2006; Havula and Hietakangas, 2012, 2018) binding site, and all sequences had one of the elements responsible for regulating genes as a response to cellular glucose levels (*Mlx* and *Mlxip*) (Stoeckman et al., 2004; Havula and Hietakangas, 2012; Havula et al., 2013; Mattila et al., 2015). *Mfsd1* was found to have the highest number of copies of the *Mlx*, *Mlxip* and *Mlxipl* binding motifs.

In order to monitor if the high prevalence of glucose and macronutrient sensing motifs affect the biological activity of these SLCs of MFS type, we looked both at the gene and protein expression of these transporters in mouse primary cultures and adult *D. melanogaster* subjected to different glucose concentrations. Cortex cultures from e15 embryos were set up, and after 9 days of culture, D-glucose was removed from the media (glucose starvation) for 3 or 12 h before RNA, protein and ICC samples were collected. In addition, two groups of primary cortex cultures subjected to D-glucose starvation were refed glucose, at standard media concentration of 4.5 g/l, for 12 h after their endpoint of starvation. The primary cortex cultures used were a heterogeneous mixture of cells, even though the culture protocol used favors neurons. The cellular expression of some of the new SLCs and putative transporters included in the study were not known to be neuronal or glial and hence a mixed culture was used. In addition, presence of astrocytes is known to influence glucose metabolism and function as a buffer for the neurons in times of hypoglycemia (Falkowska et al., 2015), and their presence in the cultures were verified. GFAP-positive cells were found in the culture, Supplementary Figure 1A, but were a minority among the cells where most cells stained positive for Pan neuronal cocktail.

Adult male flies were likewise subjected to starvation and refeeding and changes in gene expression for the SLCs were monitored. Due to the high degree of conservation found, *D. melanogaster* provide a good model for a whole biological system. Starvation is a biologically drastic state for both cell cultures and files, and to gain more precise information about glucose sensing, primary cortex cell cultures were also subjected to low glucose levels, at 1g/mL, and fruit flies were placed on different diets consisting of varying levels of sugars.

### Glucose Starvation Alters mRNA Expression of Both Several Transporters and Metabolic Targets With Most Alteration Restored After Refeeding

**FIGURE 3:**
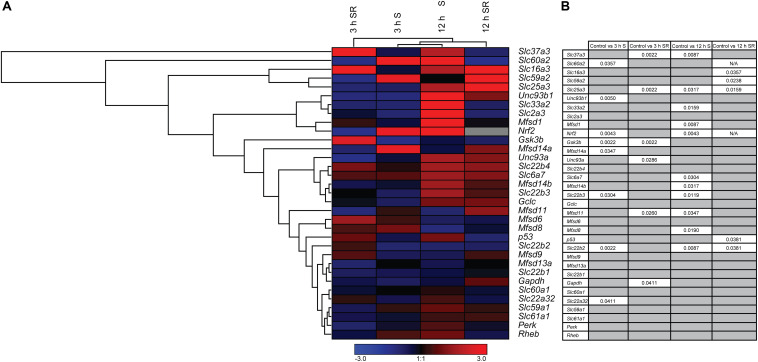
The mRNA expression regulation of putative SLCs and genes connected to glucose metabolism and stress after glucose starvation as well as glucose starvation and refeeding. Primary cortex cells from mice were either subjected to glucose starvation (S) or glucose starvation and refeeding (SR) for 3 and 12 h. The mRNA expression was measured using qRT-PCR (*n* = 5 or 6 per group) and the expression was normalized against five stable housekeeping genes. The control for each group was set to 1 and the mRNA expression for each group is relative to its control. GraphPad Prism version 5 was used to calculate differences in mRNA expression using Mann–Whitney (* > 0.05, ** > 0.01, *** > 0.001). The heatmap was generated using GENESIS version 1.7.6 using the differences in fold change between the experimental groups and controls; red = upregulation, blue = downregulation and gray = data missing. **(A)** The heatmap display the alteration in gene expression for 3 h S, 12 h S, 3 h SR and 12 h SR compared with its corresponding control in the primary cortex cells. The genes and experiments were hierarchical clustered. **(B)** The table summarize the *p*-values. For starved and refed samples of *Slc60a2* and *Nrf2*, not enough replicates were successfully analyzed to perform statistics, indicated by “N/A” in the table.

New SLCs and putative transporters were analyzed for gene regulation, together with a selection of known transporters and general metabolic targets. In total 33 different targets were analyzed using qPCR and the results are depicted in a heat map, Figure 3A, with details in Supplementary Figures 2–4. Both upregulation and downregulation were seen for transporters and metabolic targets. Six putative transporters, *Slc22a32*, *Slc22b2*, *Slc22b3*, *Unc93b1*, *Mfsd14a*, and the known glucose transporter *Slc60a2*, responded to glucose starvation already after 3 h, Figure 3B. All putative transporters returned to the same expression levels as controls after refeeding by changing to media containing 4.5 g/l of glucose. Refeeding did also alter expression of three additional putative transporters (*Slc37a3, Unc93a*, and *Mfsd11*) and the mitochondrial transporter, *Slc25a3* that were not altered by 3 h starvation.

The total number of genes responding to 12 h of starvation was higher. Nine putative transporters (*Slc22b2*, *Mfsd8*, *Mfsd11*, *Slc22b3*, *Slc33a2*, *Slc37a3*, *Mfsd1*, and *Mfsd14b*), and two known transporters, *Slc6a7, Slc25a3* showed changes in expression levels, Figure 3B. Of these, *Slc22b2* and *Slc22b3* where the only ones affected both by 3 h and 12 h starvation with *Slc22b2* downregulated at both time points and *Slc22b3* downregulated at 3 h and upregulated after 12 h of starvation. While most reverted back to control levels after refeeding with glucose, *Slc22b2* stayed downregulated even after refeeding and *Slc25a3* stayed upregulated. Refeeding 12 h starved cells did upregulate expression of one additional putative transporter, *Slc59a2*, as well as a lactate transporter, *Slc16a3*.

In addition to SLCs and putative transporters, more general metabolism and stress markers were monitored: Glyceraldehyde-3-phosphate dehydrogenase (*Gapdh*), an important enzyme in glycolysis and energy production (Ramzan et al., 2013; Seidler, 2013), Glycogen synthase kinase 3b (*Gsk3b*), plays an important role in glycogen metabolism and insulin sensing (Cohen and Goedert, 2004), Ras homolog enriched in brain (*Rheb*) involved in energy sensing (Dibble and Cantley, 2015), NF-E2-related factor 2 (*Nrf2*), a master regulator in the antioxidant response system (Itoh et al., 1997; Huang et al., 2015), Tumor suppressor p53 (*p53*) regulates biological processes in response to stress (Madan et al., 2011; Liu et al., 2015; Humpton and Vousden, 2016), Glutamate cysteine ligase, catalytic subunit (*Gclc*) rate-limiting enzyme responsible for the production of glutathione (GSH) involved in antioxidant defense (Lu, 2009; Sikalidis et al., 2014), the eukaryotic translation initiation factor 2-alpha kinase 3 (*Perk*) (Shi et al., 1998; Cullinan et al., 2003), the alpha subunit of the eukaryotic translation-initiation factor 2 (EIF2) important for protein synthesis, the neuronal glucose transporter *Slc2a3* (Nagamatsu et al., 1994; Fladeby et al., 2003), *Slc6a7*, a proline transporter (Nuttall et al., 2004; Pramod et al., 2013), a monocarboxylate transporter known to transport lactate *Slc16a3* (Halestrap, 2013), and *Slc25a3*,a mitochondrial phosphate carrier (Baseler et al., 2012). Of these, *Gsk3b* and *Nrf2* were affected at 3 h of glucose starvation, and while *Nrf 2* reverted to control levels, *Gsk3b* stayed downregulated after glucose was refed to the culture. *Gapdh* was affected not by starvation, but by refeeding 3 h starved cells. After 12 h of starvation, *Nrf2*, was once again upregulated, while no mRNA expression could be found after refeeding, indicated by the gray box in Figure 3A. Refeeding cells after 12 h of starvation downregulated *p53.*

To ensure that glucose starvation and refeeding affected the metabolism in the primary cortex cultures, total mTOR was measured after 3 and 12 h glucose starvation and after refeeding for 12 h. During starvation for 3 and 12 h, the total mTOR protein expressions were downregulated and after the cells were refed with D-glucose, the expression of total mTOR went back to normal, Supplementary Figure 1B.

### Glucose Starvation Causes Protein Expression of SLCs of MFS Type to Be Altered in Neurons

**FIGURE 4:**
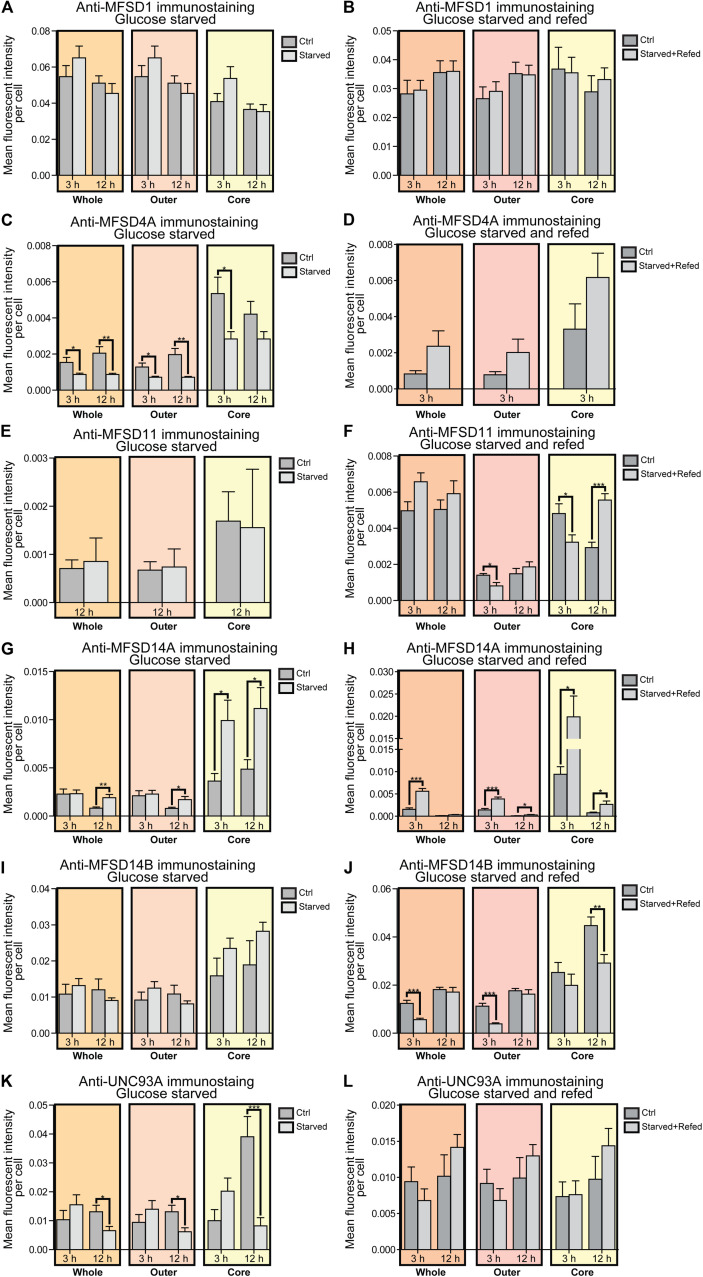
Changes in protein expression of putative SLCs subjected to glucose starvation and glucose starvation and refeeding. Primary cortex cultures subjected to glucose starvation (S) and glucose starvation and refeeding (SR) for 3 and 12 h were used for immunocytochemistry (ICC). In total, six putative SLCs were investigated where at least one antibody has been verified previously and shown to work on primary cortex cultures; MFSD1, MFSD4A, MFSD11, MFSD14A, MFSD14B and UNC93A. Images were taken using the same exposure settings for all pictures for each transporter. For each group, 6–10 pictures were taken including several cells. The images were processed in CellProfiler version 2.2.0th Identification of cellular compartments, nuclei (based on DAPI staining) and whole neuronal cytoplasm (based on Pan-neuronal staining) were performed. Mean and differences (±SEM), were calculated using unpaired *t*-test (* > 0.05, ** > 0.01, *** > 0.001). Each graph contain data regarding the mean fluorescent intensity for whole (orange), outer (apricot), and core (yellow) staining. No difference in expression was observed for MFSD1 after **(A)** glucose S nor **(B)** glucose SR. **(C)** MFSD4A was found to be downregulated in all three compartments after 3 and 12 h S, except for the core compartment after 12 h. **(D)** No alteration was found after 3 h SR, no data was collected for 12 h samples in the same experiment. No difference in immunostaining was observed for MFSD11 after **(E)** 12 h S, no data collected for 3 h S. **(F)** Reduction in immunostaining was observed in the outer and core compartment after 3 h SR, and increased immunostaining was found after 12 h SR. **(G)** The immunostaining of MFSD14A was induced in the core compartment after 3 h S, while induced in all three compartments after 12 h S. **(H)** Meanwhile after 3 h SR, MFSD14A staining was increased after 3 h in all three compartments, and still increased in the outer and core compartments after 12 h SR. **(I)** No differences were found for MFSD14B after glucose starvation, while **(J)** the immunostaining was reduced after 3 h SR in the whole and outer compartments, and after 12 h SR in the core. **(K)** UNC93A immunostaining was reduced after 12 h S in all three compartments, while **(L)** no differences were observed in cells subjected to 3 and 12 h SR.

**FIGURE 5:**
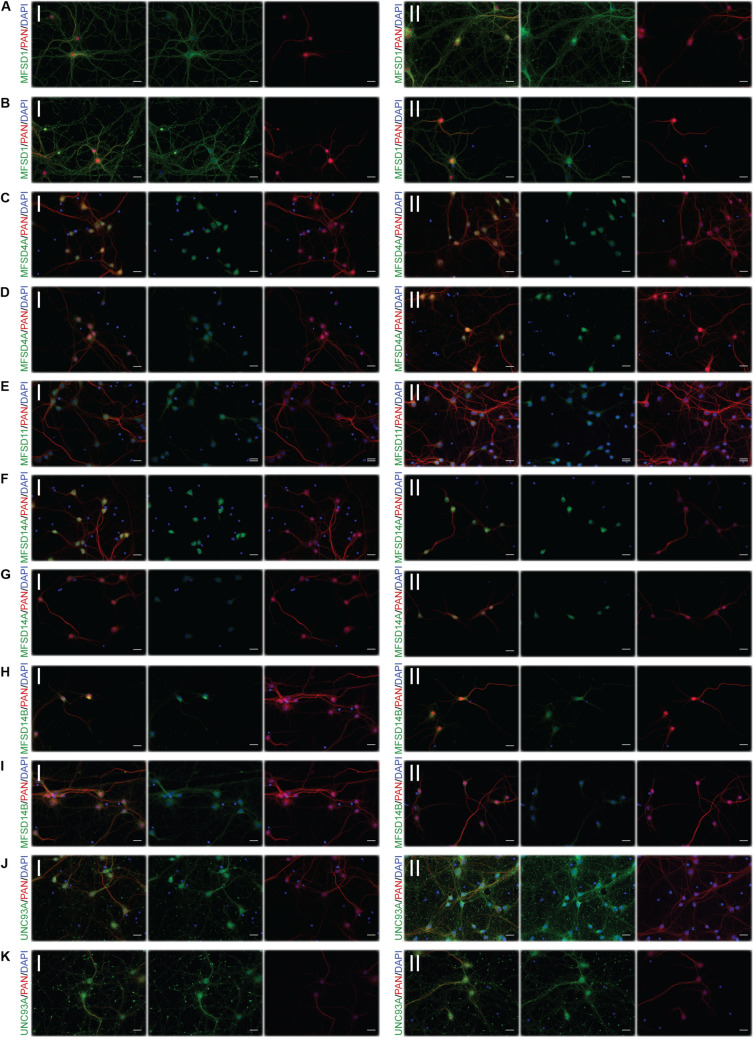
Representative ICC images of the putative SLCs subjected to glucose starvation used for measuring changes in protein expression. Primary cortex cultures subjected to glucose starvation (S) for 3 and 12 h were used for immunocytochemistry. In total, six putative SLCs, where one antibody has been verified previously and shown to stain primary cortex cultures (Perland et al., 2016, 2017a,b,c; Ceder et al., 2017; Lekholm et al., 2017), were investigated; MFSD1, MFSD4A, MFSD11, MFSD14A, MFSD14B, and UNC93A. Images were taken using the same exposure settings for all pictures for each transporter, scale bar represents 20 μm. For each group, 6–10 pictures were taken including several cells. The putative SLC is labeled in green (FITC), the neuronal marker PAN in red (cyto) and the nucleus marker (DAPI) in blue. The panel consists of the representative ICC images of each target for both the (I) control and the (II) S condition; **(A)** MFSD1 3 h, **(B)** MFSD1 12 h, **(C)** MFSD4A 3 h, **(D)** MFSD4A 12 h, **(E)** MFSD11 12 h, **(F)** MFSD14A 3 h, **(G)** MFSD14A 12 h, **(H)** MFSD14B 3 h, **(I)** MFSD14B 12 h, **(J)** UNC93A 3 h, and **(K)** UNC93A 12 h.

**FIGURE 6:**
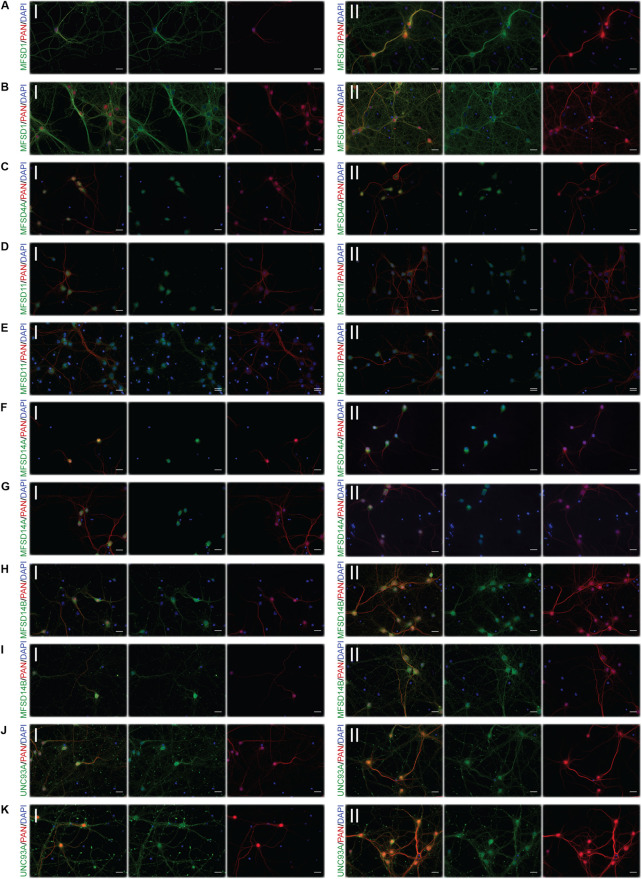
Representative ICC images of the putative SLCs subjected to glucose starvation and refeeding used for measuring alterations in protein expression. Primary cortex cultures subjected to glucose starvation and refeeding (SR) for 3 and 12 h were used for immunocytochemistry. Six putative SLCs were stained for: MFSD1, MFSD4A, MFSD11, MFSD14A, MFSD14B, and UNC93A. Images were taken using the same exposure settings for all pictures for each transporter, scale bar represents 20 μm. For each group, 6–10 pictures were taken including several cells. The putative SLC is labeled in green (FITC), the neuronal marker PAN in red (cyto) and the nucleus marker (DAPI) in blue. The panel consists of the representative ICC images of each target for both the (I) control and the (II) SR condition; **(A)** MFSD1 3 h, **(B)** MFSD1 12 h, **(C)** MFSD4A 3 h, **(D)** MFSD11 3 h, **(E)** MFSD11 12 h, **(F)** MFSD14A 3 h, **(G)** MFSD14A 12h, **(H)** MFSD14B 3h, **(I)** MFSD14B 12 h, **(J)** UNC93A 3 h, and **(K)** UNC93A 12 h.

To monitor if the glucose starvation resulted in any protein expression or localization changes, ICC was performed for those targets for which there were verified antibodies available, Figures 4–6. As to not introduce bias in the evaluation of fluorescent signal and to be able to analyze a large sample size, a pipeline in CellProfiler was used to evaluate changes in fluorescent intensities and position in the cell compared to DAPI (nucleus staining) and Pan neuronal marker, staining soma, axons and dendrites of neurons. Fluorescent intensity in the cytoplasm was compared between control and starved or control and refed, Figure 4, and representative ICC images used during the analysis are found in Figures 5, 6. The transporters that were monitored were all found in neurons. The staining was monitored in the whole cell, the outer perimeter (including projections) and the core soma (excluding nucleus).

For MFSD1, where mRNA upregulation could be seen after 12 h of glucose starvation, no alteration in protein expression was found to have taken place yet, while for MFSD4A (*Slc60a1*), protein expression was downregulated in the whole cell at both 3 and 12 h, Figure 4C, while no change in mRNA expression was seen at the same time point, Figure 3. MFSD11 staining intensity remained the same in the whole cell, however, a localization shift was seen after starvation and refeeding after both 3 and 12 h, Figure 4F. MFSD14A, which was transcriptionally upregulated after 3 h of starvation, had increased staining at 12 h in whole cell as well as the core and outer portions of the cell. Starving and refeeding glucose to cells strongly increased protein expression of MFSD14A in the whole, core and outer portions of the cells, Figures 4F,G. Refeeding glucose to starved cells likewise affected MFSD14B compared with controls, but in the opposite direction, with lower fluorescent intensity in the whole and outer portion of the cells at 3 h, and the core of the cell at 12 h, Figure 4J. Staining for UNC93A was lower after 12 h of starvation in all areas of the cells, while staining was at the same levels as controls after refeeding, Figures 4K,L.

### Starvation Affect the Transporter Orthologous in *D. melanogaster*

**FIGURE 7:**
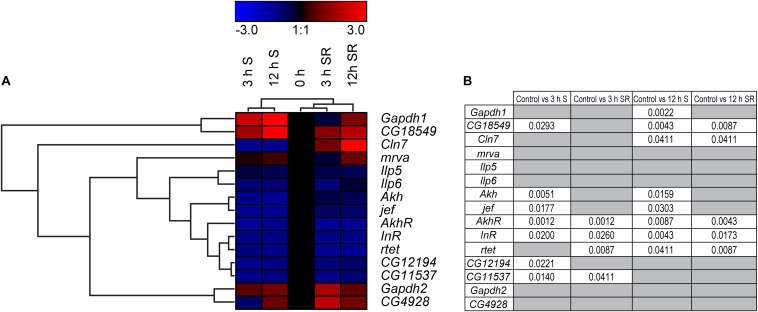
Gene regulation of putative SLCs and genes involved in metabolism in *D. melanogaster.* Four groups of adult male flies, aged 5 days, were subjected to starvation for 0 h (control), 3 and 12 h, two of the groups (*n* = 6–8, with 10 flies in each) were euthanized immediately after starvation, while two groups (*n* = 8, with 10 flies in each) were refeed with Jazz Mix Standard food for 12 h before euthanized. Samples were used to measure the mRNA expression via qRT-PCR and the expression was normalized against three stable housekeeping genes. The control was set to 1 and the mRNA expression for each group is relative to the control group (0 h of starvation). GraphPad Prism version 5 was used to calculate differences in mRNA expression using Kruskal–Wallis with Mann–Whitney as a post-hoc test with Bonferroni’s correction (*p < 0.0489, **p < 0.00995, ***p < 0.00099). The heatmap was generated using GENESIS version 1.7.6 using the differences in fold change between the experimental groups and the control; red = upregulation, blue = downregulation, black = no change. The genes and experiments were hierarchical clustered. **(A)** The heatmap display alterations in gene expression for 0 h S, 3 h S, 12 h S, 3 h SR and 12 h SR flies. **(B)** The table summarize the *p*-values.

To monitor if changes in gene expression also occurs as an effect of sugar availability in a multicellular model organism, we turned to the well-studied *D. melanogaster* (Jennings, 2011). Not all putative SLCs included in the study were conserved in flies, however, a few of them had a clear orthologue to the human and mouse sequences: *Mfsd1* (*mrva* and *CG12194*), *Mfsd6* (*jef*), *Mfsd8* (*Cln7*), *Slc22a32* (*rtet*), *Mfsd11* (*CG18549*), *Mfsd14a* (*CG11537*), and *Unc93a* (*CG4928*). General markers known to be involved in metabolism and sugar pathways were also studied; *Akh*, *AkhR* (Galikova et al., 2015), *Gapdh1*, *Gapdh2* (Sun et al., 1988; Wojtas et al., 1992), *Ilp5* (Semaniuk et al., 2018), *Ilp6* (Bai et al., 2012) and *InR* (Chen et al., 1996; Li et al., 2014), Figures 7A,B and Supplementary Figure 7. Adult male flies were subjected to 0 h (controls), 3 h and 12 h of starvation as well as refeeding after 3 h and 12 h of starvation. A majority of the SLC and putative SLC orthologues, as well as metabolic targets (*CG18549, Akh*, *jef, AkhR*, *InR*, *CG12194*, and *CG11537*) were affected after 3 h of starvation and were mostly downregulated except for *CG18594* which was upregulated. Refeeding returned the expression back to normal for *CG18549, jef* and *CG12194*, as well as for metabolic target *Akh*. Two targets, *AkhR* and *InR*, both receptors involved in glucose homeostasis, remained downregulated even after refeeding the starved flies. Eight targets were affected by 12 h of starvation, and only two *Gapdh1* and *CG18594* were upregulated, while the rest were downregulated. Refeeding starved flies after 12 h of starvation caused normalization of *Gapdh1, Akh* and *jef.* Meanwhile *AkhR*, *InR* and *rtet* were still downregulated after refeeding, and *CG18549* still upregulated. *Cln7* switched from being downregulated after 12 h of starvation to upregulated after refeeding, compared with the control. No significant changes in expression were found for *Ilp5*, *Ilp6*, *Gapdh2*, *mrva*, and *CG4928*, Figure 7. For four of the new SLCs and putative transporters, a response was seen both in glucose starved cell cultures and starved adult flies. *Mfsd1 (CG12194*), *Mfsd8 (Cln7), Mfsd11 (CG18549)*, and *Mfsd14a* (orthologue *CG11537*) were all affected by starvation, summary in Figure 12A.

### Primary Cultures Subjected to Glucose Deprivation Alter Gene Expression of Several Transporters of MFS Type

Glucose starvation of cortex cultures yielded changes in expression for some of the new SLCs and putative transporters, but not all, and by far fewer than were expected based on the presence of glucose sensing motifs. Total glucose starvation is a high stress metabolic state for cell cultures that rely heavily on glucose for energy. Likewise, total starvation for adult flies represents a high stress state where not only glucose metabolism is affected. To focus more on regulation based on glucose availability, gene regulation changes were monitored under conditions of low glucose (1g/L) in cell cultures and in flies fed both low and high sugar foods.

**FIGURE 8:**
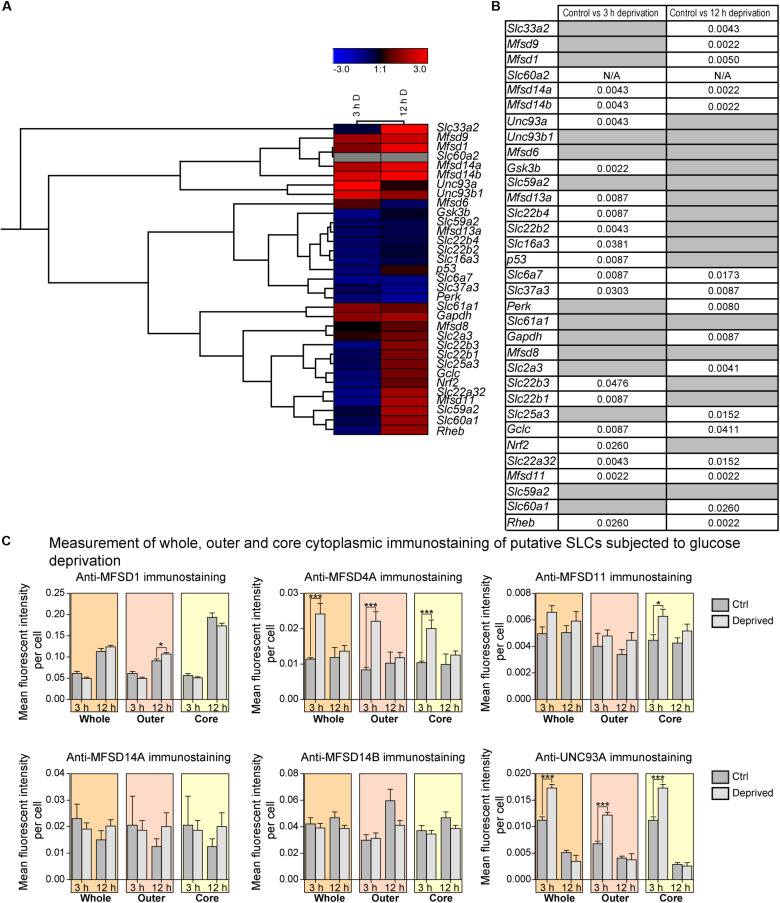
The mRNA and protein expression regulation of putative SLCs and genes connected to glucose metabolism and stress after glucose deprivation. Primary cortex cells from mice were subjected to glucose deprivation (D) for 3 and 12 h. The mRNA expression was measured using qRT-PCR (*n* = 6 per group) and the expression was normalized against five stable housekeeping genes. The control for each group was set to 1 and the mRNA expression for each group is relative to its control. GraphPad Prism version 5 was used to calculate differences in mRNA expression using Mann–Whitney (* > 0.05, ** > 0.01, *** > 0.001). The heatmap was generated using GENESIS version 1.7.6 using the differences in fold change between the experimental groups and controls; red = upregulation, blue = downregulation, black = no change and gray = data missing. The genes and experiments were hierachical clustered. **(A)** The heatmap display alterations in gene expression for 3 and 12 h glucose deprived cells compared with its corresponding control. **(B)** The table summarize the p-values. Primary cortex cultures subjected to glucose deprivation for 3 and 12 h were used for immunocytochemistry (ICC). Six putative SLCs were investigated; MFSD1, MFSD4A, MFSD11, MFSD14A, MFSD14B, and UNC93A. Images were taken using the same exposure settings for all pictures. For each group, 6–10 pictures were taken. The images were processed in CellProfiler version 2.2.0. Identification of cellular compartments, nuclei (based on DAPI staining) and whole neuronal cytoplasm (based on Pan-neuronal staining) were performed. The mean pixel intensity in protein staining was measured in each subcellular compartment and the average fluorescent intensity per cell was calculated. GraphPad Prism version5 was used to generate graphs, mean and differences (±SEM), were calculated using unpaired *t*-test (* > 0.05, ** > 0.01, *** > 0.001). Each graph contain data regarding the mean fluorescent intensity for whole (orange), outer (apricot), and core (yellow) staining. **(C)** No differences in immunostaining was observed for MFSD1, MFSD11, MFSD14A and MFSD14B, while the immunostainings for MFSD4A and UNC93A were induced in all three compartments after 3 h glucose deprivation.

In primary cortex cultures, glucose deprivation affected more than double the targets than glucose starvation did. Eighteen of 33 targets were affected after 3 h and 16 targets after 12 h of glucose deprivation. *Mfsd14a, Mfsd14b*, and *Unc93a* were upregulated at 3 h of deprivation, while *Gsk3, Mfsd13a, Slc22b4, Slc22b2, Slc16a3, p53, Slc6a7, Slc37a3, Slc22b3, Slc22b1, Gclc*, *Nrf2*, *Slc22a32, Mfsd11*, and *Rheb* where all downregulated, Figures 8A,B and Supplementary Figures 5, 6. After 12 h of glucose deprivation upregulated gene expression was seen for: *Slc33a2, Mfsd9, Mfsd1, Mfsd14a, Mfsd14b, Gapdh*, *Slc25a3, Gclc, Slc22a32, Mfsd11, Slc60a1, Rheb*, and the important neuronal glucose transporter *Slc2a3.* Downregulation was seen for just three targets, *Slc6a7, Slc37a3* and *Perk.*

A few new SLCs and putative transporter genes responded both at 3 and at 12 h, and for both *Slc22a32* and *Mfsd11*, gene expression decreased after 3 h of glucose deprivation but increased beyond controls after 12 h again. In contrast, the gene expression of both *Mfsd14a* and *Mfsd14b* significantly increased at 3 h and stayed upregulated even at 12 h, compared with controls. The known transporters, *Slc6a7* and *Slc37a3* were both downregulated at both time points. Putative SLC genes that did not respond to the glucose deprivation in the media were: *Unc93b1*, *Mfsd6, Slc59a2, Slc61a1, Mfsd8*, and *Slc59a2*, Figure 8, and overview in Figures 11, 12.

### Protein Expression of Some SLCs of MFS Type Are Altered Depending on the D-Glucose Availability

**FIGURE 9:**
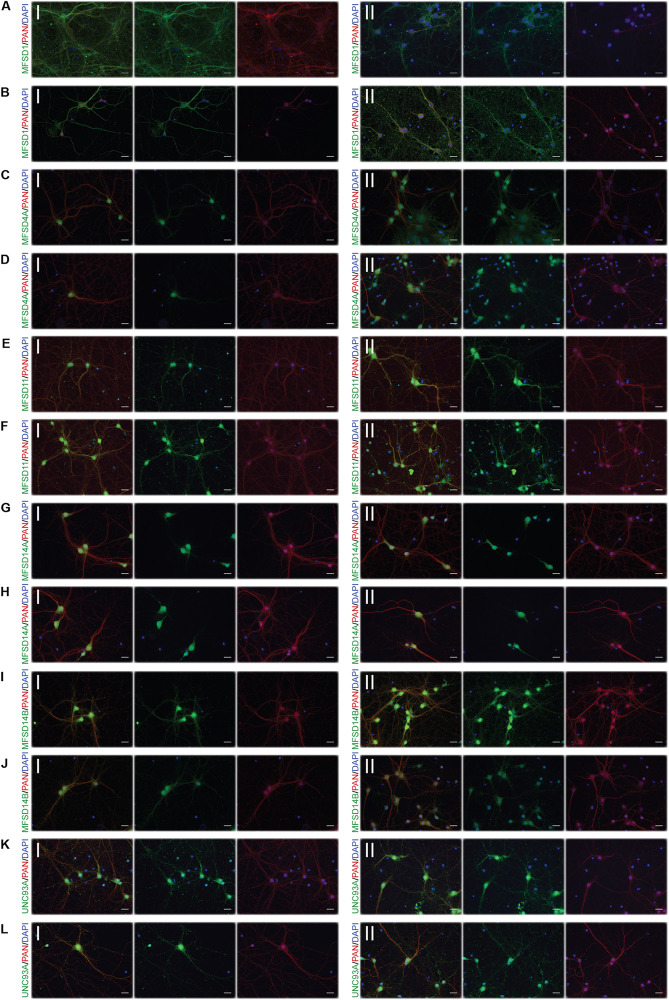
Representative ICC images of the putative SLCs subjected to glucose derpivation used for measuring changes in protein expression. Primary cortex cells from mice were subjected to glucose deprivation (D) for 3 and 12 h were used for immunocytochemistry. MFSD1, MFSD4A, MFSD11, MFSD14A, MFSD14B, and UNC93A were stained for. Images were taken using the same exposure settings for all pictures for each transporter, scale bar represents 20 μm. For each group, 6–10 pictures were taken including several cells. The putative SLC is labeled in green (FITC), the neuronal marker PAN in red (cyto) and the nucleus marker (DAPI) in blue. The panel consists of the representative ICC images of each target displaying both the (I) control and the (II) glucose deprivation; **(A)** MFSD1 3 h, **(B)** MFSD1 12 h, **(C)** MFSD4A 3 h, **(D)** MFSD4A 12 h, **(E)** MFSD11 3 h, **(F)** MFSD11 12 h, **(G)** MFSD14A 3 h, **(H)** MFSD14A 12h, **(I)** MFSD14B 3h, **(J)** MFSD14B 12 h, **(K)** UNC93A 3 h, and **(L)** UNC93A 12 h.

For a few of the putative transporters, antibodies yielding good quality staining were found and fluorescent intensity was again monitored using the CellProfiler pipeline, Figure 8C, with representative ICC images used for the analysis in Figure 9. MFSD1 expression, which had an upregulated gene expression after 12 h of deprivation, was seen to increase in the outer portions of neurons after 12 h of glucose deprivation, Figure 8C. MFSD4A, also with an increased gene expression (*Slc60a1)* after 12 h of deprivation, had higher fluorescent staining in the whole, outer and core parts of the cells already after 3 h. MFSD11 staining was found to increase in the core of neurons after 3 h of deprivation, as did the expression of UNC93A. UNC93A staining was also seen to increase in the whole and outer parts of the neurons after just 3 h of deprivation, mirroring the increased gene expression seen at 3 h. For both MFSD14A and MFSD14B, no change in protein expression could be seen, Figure 8C, while the gene expression was increased for both transporters at both time points, Figure 8A.

### Deprivation and High Intake of Sugar Affect the Transporter Orthologous in *D. melanogaster*

**FIGURE 10:**
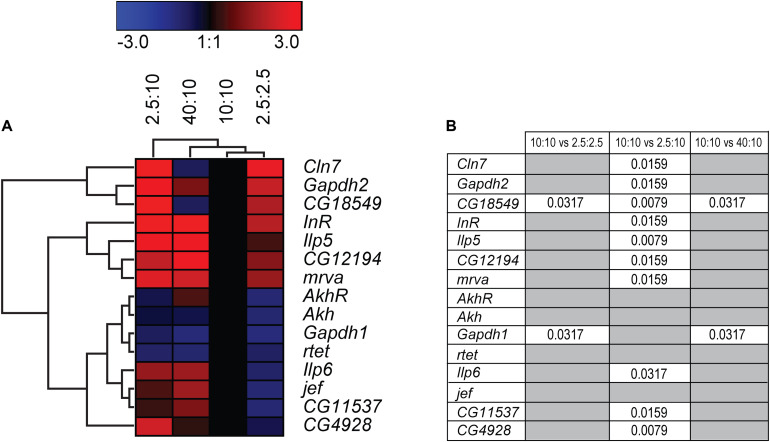
Gene regulation of putative SLCs and metabolic genes after feeding fruit flies different diets. Adult male flies (*n* = 6 with 10 flies in each), aged 5 days, were maintained for 5 days on low calorie (2.5 g:2.5 g sugar:yeast), low sugar (2.5 g:10 g sugar yeast), normal sugar (10 g:10 g sugar:yeast) (control), and high sugar (40 g:10 g sugar:yeast) diets. The mRNA expression was measured via qRT-PCR and the expression was normalized against three stable housekeeping genes. The control was set to 1 and the mRNA expression for each group is relative to the control group (10:10). GraphPad Prism version 5 was used to calculate differences in mRNA expression using Kruskal–Wallis with Mann–Whitney as a post-hoc test with Bonferroni’s correction (*p < 0.0489, **p < 0.00995, ***p < 0.00099). The heatmap was generated using GENESIS version 1.7.6 using the differences in fold change between the experimental groups and the control group; red = upregulation, blue = downregulation, black = no change. The genes and experiments were hierarchical clustered. **(A)** The heatmap display the alteration in gene expression for flies subjected to low calorie, low sugar, high sugar and control diet. **(B)** The table summarize the p-values.

Focusing on effects caused by sugars and not just general starvation, adult males were feed various concentrations of sugars and yeast to mimic fluctuating sugar availability in the body. Flies were feed a normal (10:10 g/dl sugar:yeast (S:Y)), low calorie (2.5:2.5 g/dl S:Y), low sugar (2.5 g/dl S:Y) and sugar enriched (40:10 g/dl S:Y) diets for 5 days before RNA was isolated. Decreasing both sugar and yeast to a low-calorie diet, increased the expression of *CG18549* (the orthologue to *Mfsd11*), while decreasing both *Gapdh1* and *CG11537* (orthologue to *Mfsd14a)*, Figure 10. Decreasing just the sugar content while keeping the protein content at normal level, produced the highest gene expression shift seen yet, with nine of 15 targets affected and all of the genes affected were upregulated, Figure 10. Interestingly, increasing just the sugar content only affected two genes, *CG18549* and *Gapdh1*, Figures 10, 12B. *CG18549* (orthologue to *Mfsd11)* showed changed expression in all three diets compared with controls, increased in both a general low-calorie diet and a low sugar diet while decreased in the high sugar diet.

## Discussion

Here we have used two model systems to study the effects that total glucose/nutrient starvation and low/high glucose levels have on a group of SLCs and putative transporters. One model was made from mouse primary cortex cultures, and the other was adult male *D. melanogaster*. The cell cultures were composed of a mixture of cells, where the presence of both astrocytes and neurons were verified, and our findings are compared with normal culturing protocols where non-physiological concentrations of glucose (4.5 g/l) is used. However, the energy consumption of multicellular organisms is regulated by hormones and polypeptides e.g., insulin and glycogen, a tribute that the unicellular systems largely miss. Using these two systems together, information of both specific and overall regulation of these putative transporters was collected and while we did find comparable reactions in both systems for some targets, some were different. Based on the glycemic need of the neurons, most protocols suggest a glucose concentration of 4.5 g/l to sustain neuronal culture (Bottenstein and Sato, 1979; Brewer et al., 1993), hence, the cell cultures were grown in this glucose concentration until day 10 and control cells in this experimental setup was continued on 4.5 g/l of glucose and deprived using1 g/l of glucose. However, it is important to understand that the deprived condition, where the media contain 1 g/l glucose (5.5 mM), is similar to the *in vivo* physiological concentration of glucose. Interestingly, this concentration has been shown to sustain neuronal metabolism as well (Kleman et al., 2008). Therefore, the model used here to study deprivation can be questioned as comparable to *in vivo* settings (Buchakjian and Kornbluth, 2010), and instead the regulation that we observe are a comparison representing the gene and protein expression of these putative SLCs during “normal” physiological glucose concentrations and a high glucose concentration (25 mM). Importantly, the results in this paper are always compared to controls with 4.5 g/l glucose. Hence, the results might not mirror the regulation in the brain during glucose deprivation (Sunwoldt et al., 2017), but it provides information about how the gene and protein expression of putative SLCs are affected in altering glucose levels.

The newly identified SLC and putative transporters that were included here have been found to be mostly neuronal (Perland et al., 2016, 2017c; Ceder et al., 2017; Lekholm et al., 2017), and the staining found here was also localized to neurons in the mixed cultures used. Glucose is mainly used to provide the neurons with one highly important product, energy in the form of ATP, a task performed in the cytosol and within the mitochondria (Jonckheere et al., 2012). The ATP is used to fuel action potentials but also to maintain ion gradients and neuronal membrane potentials. In addition, glucose is used to both drive the production, and as a precursor for, biosynthesis of neurotransmitters (Dienel, 2012; Harris et al., 2012; Mergenthaler et al., 2013). A majority of the SLCs act as cotransporters or exchangers, where ions are needed to maintain the transport, meaning that both nutrient and ion levels affect their activity and function (Cesar-Razquin et al., 2015; Perland and Fredriksson, 2017; Zhang et al., 2018). In addition, since these reactions take place at different sites of the cells, transport through membranes are needed not just for glucose, but for additional substrates and byproducts of glucose metabolism.

**FIGURE 11:**
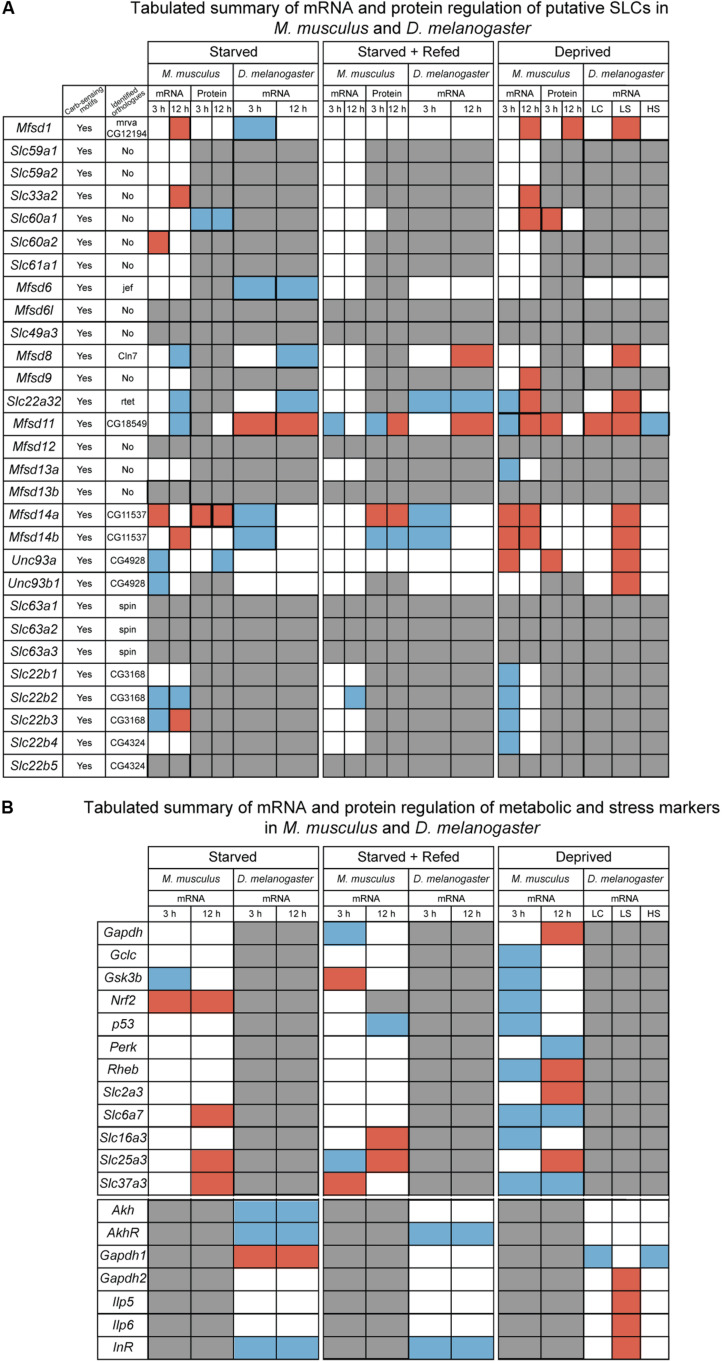
Summarizing of the results from *in vitro* and *in vivo* experiments. **(A)** Tabulated summary of results regarding the putative SLCs obtained from mouse primary cortex cultures and fruit fly; displaying the presence of carbohydrate sensing motifs (Yes/No), identified orthologues and their name, gene and protein regulation for the three diets performed in primary cultures (starved, starved+refed, and deprived), gene regulation for fruit flies subjected to different diets [starved, starved+refed, low calorie (LC), low sugar (LS) and high sugar (HS)]. Red box indicate upregulation, blue box indicate downregulation, white box illustrates no regulation observed and gray box display where information is not obtained. **(B)** Tabulated summary of metabolic and stress targets in primary cortex cells and fruit flies. Red box indicate upregulation, blue box indicate downregulation, white box illustrates no regulation observed and gray box display where information is not obtained.

**FIGURE 12:**
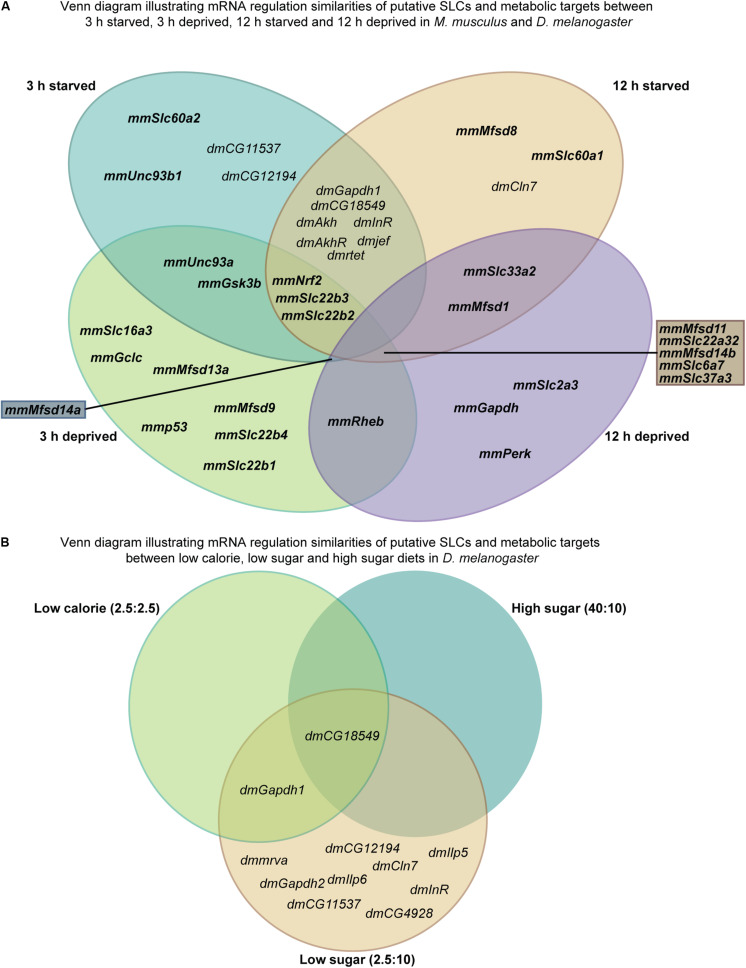
Illustrating similarities between starved and deprived of the results from *in vitro* and *in vivo* experiments. **(A)** Venn diagram displaying similarities and dissimilarities in gene regulation between starved and deprived cortex cultures and fruit fly. **(B)** Venn diagram illustrating gene regulation similarities and dissimilarities between the diets (low calorie, low sugar, and high sugar) performed in *D. melanogaster*.

Based on the gene regulation changes found in both primary cortex cultures and in adult *D. melanogaster*, subjected to no or low glucose availability, many of the newly identified SLCs and putative transporters have a potential to be involved in nutrient intake and maybe also metabolism. However, the magnitude of up- and downregulation presented in Figure 3A does not match the level of significance in Figure 3B. This is not surprising giving the rather high expression variance for some of the targets not evident in the heatmaps but affecting the conclusions that can be drawn from the gene regulation changes. A higher n number might have resolved this issue. Moreover, due to the lack of knowledge about exact cellular localization and transported substrate of many of the SLCs included in this study, more work is necessary to pin-point their exact contribution. An overview of the targets, orthologues, and gene regulation after glucose starvation, deprivation, and different diets can be found in Figures 11, 12. Not all new SLCs and putative transporters were found to be conserved in the fly, but for 14 of the targets included, one orthologue was found. Perhaps the conservation between these 14 implies a general basal function for cells. For one of these, no mRNA expression was detected in the adult flies included in our study. Similarly, some of the transporters were not found to be expressed in the mouse primary cortex cultures that were used (lack of information indicated by gray squares in Figure 11A). Equating mouse primary cortex cultures and adult flies is not a balanced comparison, but it does reveal that some transporters, and putative transporters, do react in a similar fashion. This was especially apparent when comparing glucose deprived primary cortex culture and the low sugar diets in the flies.

**FIGURE 13:**
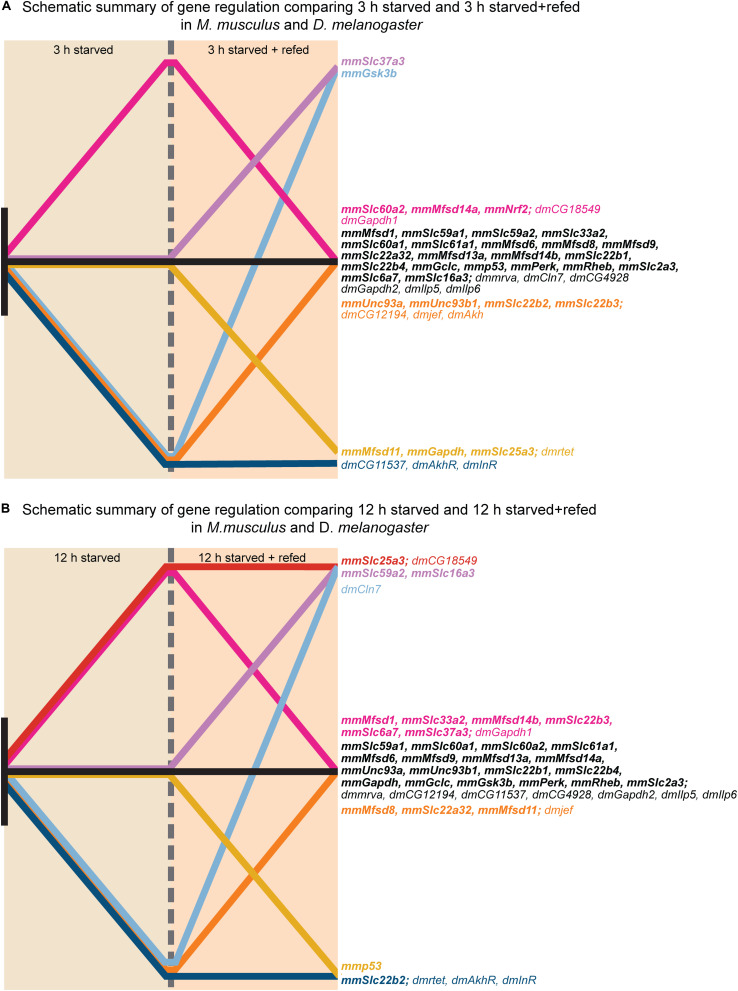
Graphical illustration of gene regulation in *in vitro* and *in* vivo. The gene regulation for each putative SLC and metabolic target measured in both primary cortex cultures and fruit flies were summarized in two graphical illustrations; one for each timepoint; no regulation (black), no regulation at 12 h S and upregulation at 12 h SR (purple), upregulation for both experimental groups (red), no regulation at 12 h S and downregulation at 12 h SR (yellow), downregulation at 12 h S and no regulation at 12 h SR (orange), downregulation at 12 h S and upregulation at 12 h SR (light blue), downregulation for both experimental groups (blue). **(A)** Display gene regulation after 3 h starved and starved+refed. **(B)** Display gene regulation after 12 h starved and starved+refed.

Upregulation in cells and flies due to low glucose availability were observed for *Mfsd1* (and orthologue *CG12194*), *Slc22a32* (orthologues *rtet*), *Mfsd11* (orthologue *CG18549*), *Mfsd14a* and *Mfsd14b* (with common orthologue *CG11537)*, and *Unc93a* (with orthologue *CG4928*), Figures 7, 10, with summary in Figure 11. All of these, apart from *Mfsd11*, were only upregulated in *D. melanogaster* by the low sugar diet, not the low-calorie diet, which suggest that they are possibly regulated by factors that response to sugar levels rather than an overall low level of nutrient. The responsiveness of the putative transporters included in our study, became more evident by re-introducing glucose to the cell cultures or refeeding the flies after total starvation. A few normalized their expression to control levels after introduction of glucose, for summary see Figures 13A,B. A 3-h lack of glucose in cell media, or a complete lack of food for adult flies, followed by refeeding normalized the expression of *Slc60a2, Mfsd14a* and *CG18549* back down to normal levels. Conversely, *Unc93a, Unc93b1, Slc22b2, Slc22b3, CG12194* and *jef* were all downregulated and then restored after refeeding. This indicates a rather quick and dynamic modulation due to energy availability. After 12 h of starvation and subsequent refeeding, *Mfsd1, Slc32a2, Mfsd14b, Slc22b3, Slc6a7*, and *Slc37a3* were all upregulated and then stabilized. *Mfsd8, Slc22a32, Mfsd11* and *jef* were instead downregulated and then returned to normal levels.

From predictions of modulatory binding sites upstream of the transcription site, Figure 2*missing*, Mfsd1 was predicted to contain one of the highest number of carbohydrate sensing motifs docking sequences, with over nine sites of *Mlx*, *Mlxip* and *Mlxipl*, key regulators of metabolic adaptations to glucose (Stoeckman et al., 2004; Havula and Hietakangas, 2012, 2018; Havula et al., 2013) and was one of the putative transporters that reacted to total lack of glucose in the cell media as well as starvation in the flies where the *Mfsd1* orthologue, *mrva*, has recently been found to be a key regulator for O-glycosylation of proteins (Valoskova et al., 2019). The other transporters seen to modulate their gene expression likewise had predicted carbohydrate sensing motifs in their promotor regions, though not as numerous as *Mfsd1*. The cellular localization of MFSD1 are contradicting, studies done in mice found it to be a lysosomal protein (Massa Lopez et al., 2019), while Perland et al. (2017b) identified MFSD1 to be a plasma membrane bound protein in primary cortex cells from mice. MFSD1 has been found to be tightly connected to Glycosylated Lysosomal Membrane Protein (GLMP) where the loss of MFSD1 causes severe liver disease in MFSD1-knockout mice. Despite the clear physiological effect of the loss of *Mfsd1*, the precise function or transported substrate was not found even though several different amino acids were tested (Massa Lopez et al., 2019). *Slc22a32* (previously named *Mfsd10* before inclusion into the *Slc22* family) was also one of the strongly regulated targets in our study and has an unknown function but a structure that resembles that of other transporters. Recently, *Mfsd10* has been identified as a protein expressed at the nuclear envelope, possibly involved in transport of toxic material out from the nuclear environment (Cheng et al., 2019). In our experiments, *Slc22a32 (Mfsd10)* was downregulated during glucose starvation in cortex cells, and in starved adult flies, while upregulated during glucose deprivation in both cells and flies. *Mfsd11* (*CG18549*) was the only target upregulated by both a low sugar and low-calorie diet in the flies, and conversely, downregulated by a high sugar diet. *Mfsd11* was also predicted to have the highest number of gene modulation sites associated with whole body energy, Figure 2. Not much is known about MFSD11, however, in a study performed using the mouse hypothalamic cell line, N25/2, *Mfsd11* was seen to be upregulated by serum and amino acid starvation (Hellsten et al., 2017), while in our setting, *Mfsd11* expression was decreased after 12 h of glucose starvation. A general role in metabolism is a high probability. Recently, *MFSD11* was identified as a novel candidate linked to intellectual disability (Anazi et al., 2017). *Mfsd6* (with orthologue *jef*), was the only target that was not altered in our primary cortex cultures but downregulated in starved adult flies. *Mfsd6* has been found to be downregulated in whole brain slices from mice fed a high calorie diet as well as in brains from mice placed on starvation (Bagchi et al., 2020), so using primary cortex cultures to study this putative transporter is likely not the best option. While *Mfsd6* expression was found in our cells, other factors available in a whole biological system that are not present in a cell culture are needed for its regulation. However, it might also be un-affected by glucose availability, but rather depend on another macronutrient. Two putative transporters whose expression were found to be highly dynamic in our setting were *Mfsd14a* and *Mfsd14b*, along with their common orthologue *CG11537*, Figures 11–13. They were both upregulation due to glucose starvation in cell cultures while they were both downregulated in starved flies. They were both upregulated during low glucose conditions (glucose deprivation) in the cell cultures, and after low sugar diet in the fly, while no change was seen after a low calorie or high sugar diets. The gene expression was normalized after refeeding. The function of MFSD14A and MFSD14B is unclear, however, MFSD14A has been linked to infertility in male mice due to a marked reduction of spermatozoa possibly due to faulty glycosylation (Doran et al., 2016), and both have been found to be intracellular proteins most located to the Golgi and endoplasmic reticulum, respectively (Lekholm et al., 2017). Interestingly, the protein expression of MFSD14A was upregulated in the glucose starved cells as well as after glucose refeeding. MFSD14A was the only putative transporter that is suggested to be intracellularly that displayed an increase in the whole cytoplasm, suggesting that this transporter, not only increase in expression, but also potentially move from its location. The Golgi apparatus harbors mechanisms that can sense nutrient availability and regulate cellular processes through O-GlcNAcylation of proteins, and re-stacking of the Golgi apparatus can aid in autophagy (Zhang and Wang, 2018). The increased protein expression seen for MFSD14A after starvation, in Figure 4, could be due to a restacking of the Golgi apparatus and increased autophagy taking place during starvation.

Similar gene regulation changes were seen for members of the SV2 family, *Slc22b1, Slc22b2, Slc22b3* and its relative *Slc22b4*. These genes code for synaptic vesicle glycoproteins, present on synaptic vesicle of neurons (Buckley and Kelly, 1985; Bajjalieh et al., 1994). All isoforms were seen to respond in a similar fashion during glucose deprivation in the cell cultures, where a transient downregulation was seen at 3 h and normal mRNA levels found at 12 h, perhaps due to a slower vesicle recycling needed during low glucose availability. During total glucose starvation, the major isoform *Slc22b1*, was not affected. SV2A, the protein of *Slc22b1*, is known to harbor important function during normal neurotransmission but has in recent years also been implicated as a galactose transporter (Madeo et al., 2014). SV2B (*Slc22b2*) and SV2C (*Slc22b3*) were however both affected by a total lack of glucose. The function of these two proteins is not as vigorously studied as that of SV2A, however, they also perform functions connected to synaptic transmission. An interesting feature that all four isoforms share is a possible binding site for adenine nucleotides such as ATP and NAD^+^ (Yao and Bajjalieh, 2008, 2009). ATP binding and modulating of transport function has been found for the glucose transporter Glut1 (*SLC2A1)*, where ATP binding affects transport activity (Levine et al., 2002) and SV2 proteins could also be regulated by the presence of ATP providing a connection between cellular metabolism, neurotransmission and vesicle recycling.

The putative transporter UNC93A has previously been found to react to nutrient availability, both in mice subjected to complete starvation for 24 h and in cell cultures subjected to amino acid starvation (Ceder et al., 2017). Our results suggest that UNC93A expression is also affected by glucose availability. However, the mRNA expression in both mice cell cultures and fly were not affected by starvation, but rather by deprivation (upregulated) and glucose refeeding after starvation (downregulated) in cells and flies. UNC93A has been suggested to be a regulator of Twik-related acid potassium 1 (TASK^1^) channels in *C. elegans* (de la Cruz et al., 2003), that are known to be important for the resting potential in neurons (Duprat et al., 1997, 2007; Karschin et al., 2001). The difference in regulation between no glucose and low glucose could point to this function. Hence, the upregulation during deprivation could be due to e.g., glucose transport and a reaction to insulin secretion, since these three molecules are tightly connected (Palmer and Clegg, 2016), while the downregulation after refeeding could possibly be an attempt to reduce the loss of potassium since starvation often cause the ion storage depletion and after refeeding the depleted storage is quickly used up.

Subjecting mouse embryonic cortex cultures to glucose starvation or deprivation also affected general metabolism targets, as did starvation and nutrient restriction in the flies. *Nrf2*, involved with antioxidant defense mechanisms (Itoh et al., 1997; Lu, 2009; Sikalidis et al., 2014; Huang et al., 2015) was increased during total lack of glucose, but normalized after refeeding. Glucose starvation and deprivation also downregulated general responses in pathways coupled to metabolisms, such as *Gsk3b*, important in glycogen metabolism and insulin sensing (Cohen and Goedert, 2004) while *Rheb*, involved in energy sensing (Dibble and Cantley, 2015) was only affected by glucose deprivation in the cell cultures. Also, more affected by low levels of glucose rather than none, was *Gapdh*, where an upregulation was seen after 12 h (Seidler, 2013). In *D. melanogaster*, two variants of *Gapdh* are present and *Gapdh1* was upregulated after total starvation in the adult flies, while downregulation was seen after the low calorie and high sugar diet, summary in Figure 11B. *Gapdh2*, meanwhile, was only upregulated during low sugar conditions. These alterations serve as a verification that the time points and glucose levels used in the set up are sufficiently long to elicit a response in the two models used. For the primary cortex cultures, deprivation seems to induce more targets involved in metabolism than starvation, correlating with the higher number of putative transporters also affected by this condition. A known neuronal glucose transporter that was also affected by glucose deprivation, rather than no glucose, was *Slc2a3.* The regulation of *Slc2a3* due to glucose deprivation had already been shown in primary neuronal cultures from rat embryos, where cultures deprived for 48 h caused a 4-fold increase of gene expression (Nagamatsu et al., 1994). Here, gene expression of *Slc2a3* increased after 12 h, while no difference could be seen after 3 h. For this transporter, 3 h of glucose deprivation is too short to elicit a response, while 12 h is just enough. This could well be true for other putative transporters included here, meaning that even though some transporters were not found to react in this setting, they might well be involved in glucose metabolism and regulated by it.

Several known SLCs that were monitored also increased by a lack of glucose in the cortex cell cultures, *Slc6a7, Slc25a3*, *Slc37a3*, and *Slc16a3*. These SLCs are not directly involved in glucose transport but connected to general metabolism and delivers information about how other SLCs react to no or low glucose and nutrient levels. For example, SLC6A7 transports proline, an important molecule for several cellular aspects e.g., protein synthesis and metabolism as well as antioxidative reactions and immune responses (Bagchi et al., 2020). The increase in expression observed for *Slc6a7* could be a secondary effect due to the glucose starvation i.e., the cell turns to other nutrients in an attempt to increase the energy status to maintain important functions (Buckley and Kelly, 1985; Bajjalieh et al., 1994; Li et al., 2014; Doran et al., 2016; Zhang and Wang, 2018), or it shows that the cells have altered antioxidative reactions and stress.

Our findings demonstrate how tightly connected and regulated the metabolic pathways are within both unicellular and multicellular life-forms. Several genes are regulated simultaneously maybe to activate alternative metabolic pathways as a response to fluctuating nutrient levels or just as a response of other upstream genes. Our results establish that SLCs of MFS type are regulated by glucose availability and could be involved in several cellular aspects that are regulated by glucose and/or its metabolites. The recently classified and putative SLCs that we have monitored in this study have a probability of being necessary for normal neuronal function during fluctuating energy and glucose availability. However, establishing their exact core function during these glucose conditions remains. The fact that almost one third of transporters and transporter-related proteins remain orphans with unknown, or contradictive, location and function, establishes the need for further research about them to fully understand their mechanistic role and their impact on cellular conditions.

## Data Availability Statement

The datasets that contain protein sequences and the script presented in this study can be found in online repositories. The names of the repository/repositories and accession number(s) can be found in the article/Supplementary Material. All images used for protein expression and localization analysis can be received upon request to the corresponding author.

## Ethics Statement

The animal study was reviewed and approved by All procedures involving mice were approved by the local ethical committee in Uppsala (Uppsala Djurförsöksetiska Nämnd, Uppsala District Court, permit number C39/16, C67/13 and C419/12) in unity with the guidelines of European Communities Council Directive (2010/63).

## Author Contributions

MC planned and performed diet experiments in primary cells, RNA extractions, cDNA synthesis, primer design, qPCR, ICC and imaging, diet experiments in *D. melanogaster*, cDNA synthesis and qPCR on flies, phylogenetic and promoter analysis. Analyzed qPCR, protein expression. Compiled figures and drafted the manuscript. EL planned and performed diet experiments in primary cells, RNA extractions, cDNA synthesis, qPCR, WB, ICC, imaging, set up pipeline in Cellprofiler, aided in the analysis of qPCR results drafted the manuscript. AK set up pipeline in Cell profiler, drafted part of the manuscript. RT performed and analyzed western blot results, drafted parts of the manuscript. NS performed diet experiments in primary cultures, prepared ICC samples, aided in RNA extractions and cDNA synthesis. LW performed primer optimization, qPCR for the starved and starved+refed primary cortex cells. SP aided in fly experiments, collection of male flies and RNA extractions of fly samples. RF aided in the planning of experiments, interpretation of results, performed cell analyzing, drafted parts and proofing of the manuscript. All authors contributed to the article and approved the submitted version.

## Conflict of Interest

The authors declare that the research was conducted in the absence of any commercial or financial relationships that could be construed as a potential conflict of interest.

## Footnotes

1 http://slc.bioparadigms.org/
