# Supplementary material for: Glucose Availability Alters Gene and Protein Expression of Several Newly Classified and Putative Solute Carriers in Mice Cortex Cell Culture and D. melanogaster
Source: Front Cell Dev Biol. 2020 Jul 7;8:579. doi: 10.3389/fcell.2020.00579 (PMC7358622; doi:10.3389/fcell.2020.00579)
Supplement: Supplementary file 1 [file Data_Sheet_1.pdf]

Glucose availability alters gene and protein expression of several newly classified and putative Solute Carriers in mice cortex cell culture and D. melanogaster.

Supplementary Material

1 Supplementary Data 1, Data Sheet 2

Contain protein sequences of the newly classified SLCs and putative SLCs in human and mouse, obtained from uniport.org, as well as the protein sequences identified when using the HMMs to search for orthologues in the proteome of *D. melanogaster*.

2 Supplementary Data 2, Data Sheet 3

Contain the CellProfiler script used for image analysis.

3 Supplementary Figures

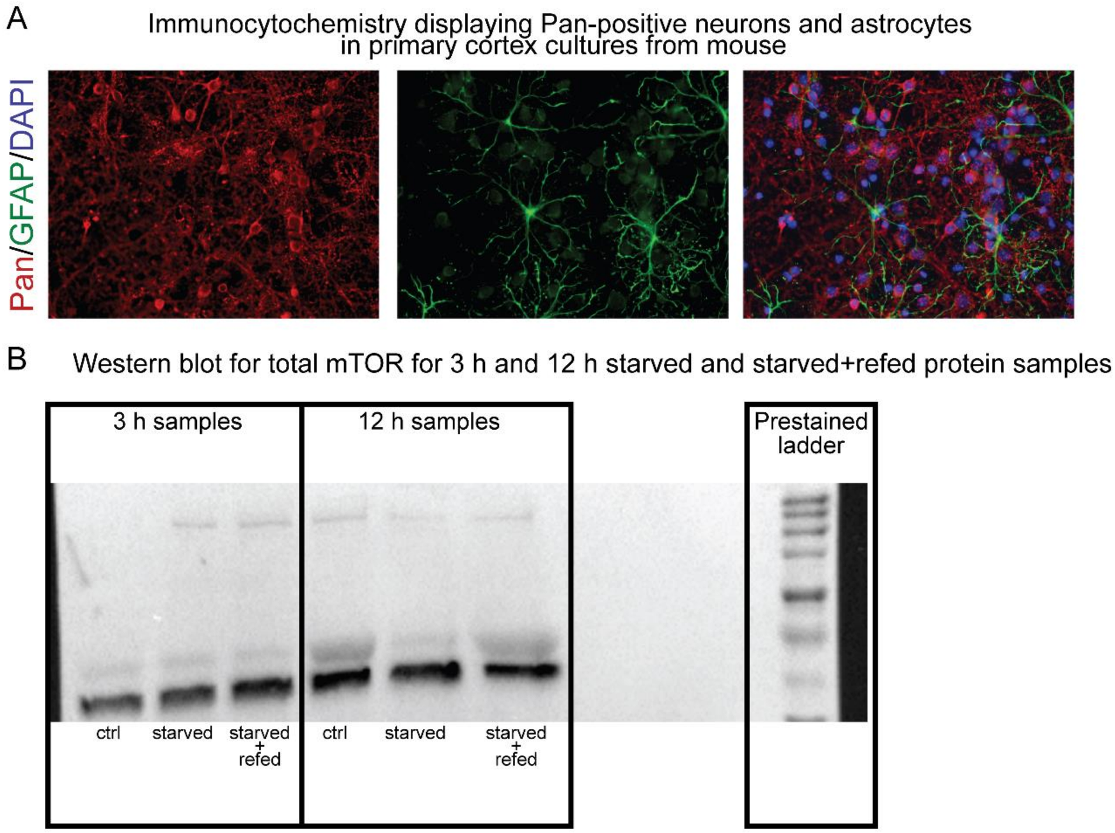

Supplementary figure 1: Immunocytochemistry and western blot on primary cortex cultures. Immunocytochemistry was performed on wild-type primary cortex cells to obtain an indication in the

distrubtion of neurons and astrocytes. Pan (red) was used as a neuronal marker, GFAP (green) was used as marker for astrocytes and the nucleus is stained with DAPI (blue). (A) the primary cortex cultures were found to contain both neurons and astrocytes. The metabolic status for primary cortex cells was measured in primary cortex cells subjected to starvation and starvation refeeding and controls. Staining for total mTOR and β-actin was performed on the same membrane. Protein samples for both timepoints (3 h and 12 h) were used. (B) Western blot membrane merged with ladder for total mTOR and β-actin.

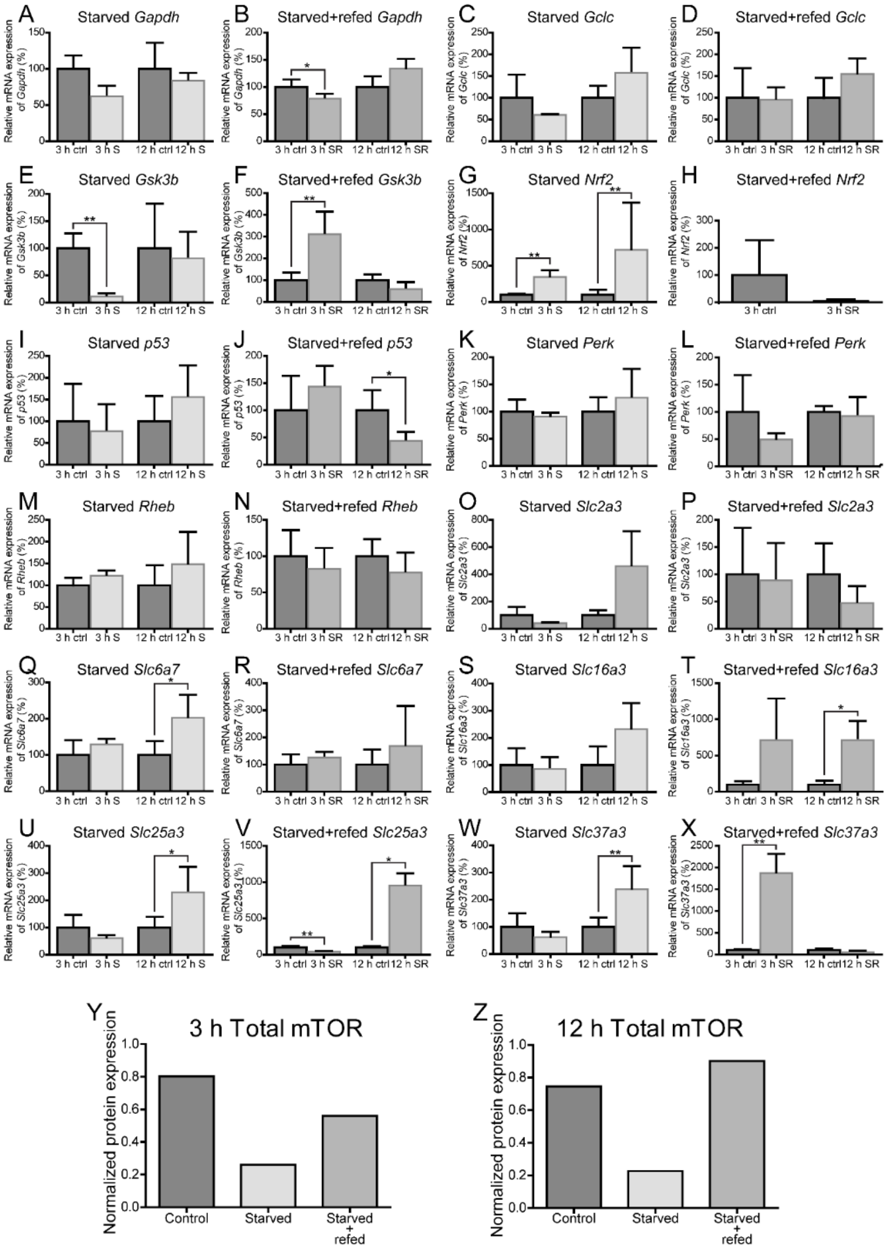

Supplementary figure 2: Relative mRNA expression and total mTOR protein expression for the metabolic and stress targets for starved and starved+refed primary cortex cultures. The mRNA expression (n=5-6) was measured using qRT-PCR and the expression was normalized against five house keeping genes. The control for each group was set to 100 %. Graphpad Prism verison 5 was used to calcualte differences in mRNA expression using Mann-whitney (*>0.05, **>0.01, ***>0.001). Graphs display gene

regulation of (AB) *Gapdh*, (CD) *Gclc*, (EF) *Gsk3b*, (GH) *Nrf2*, (IJ) *p53*, (KL) *Perk*, (MN) *Rheb*, (OP) *Slc2a3*, (QR) *Slc6a7*, (ST) *Slc16a13*, (UV) *Slc25a3* and (WX) *Slc37a3*. The total mTOR was measured using western blot. The mTOR expresison was normalized against β-actin and Graphpad Prism version 5 was used to plot the result. (Y) Total mTOR expresison in controls, 3 h starved and starved+refed primary cortex cells. (Z) Total mTOR expression in controls, 12 h starved and starved+refed primary cortex cells.

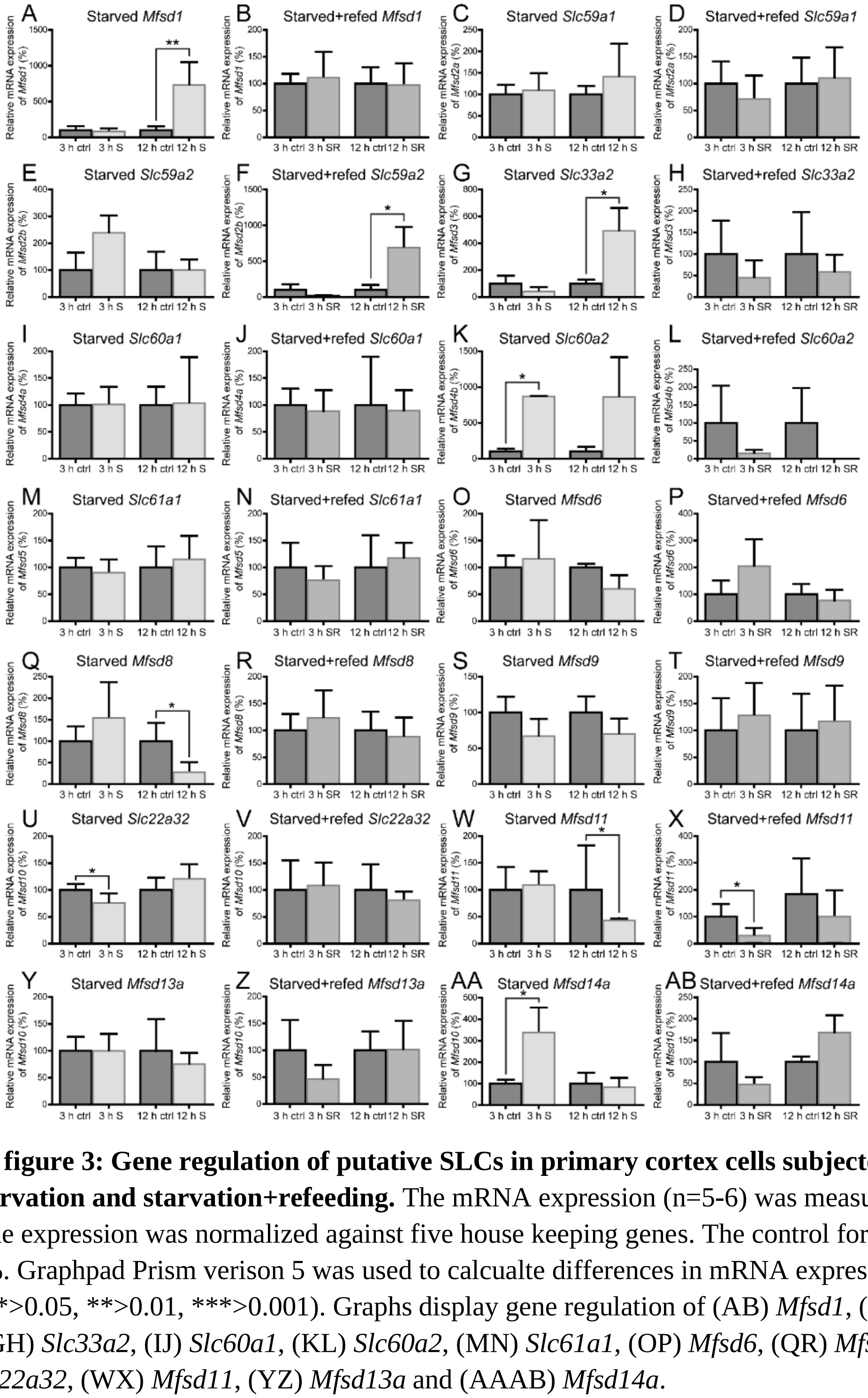

Supplementary figure 3: Gene regulation of putative SLCs in primary cortex cells subjected to 3 h and 12 h glucose starvation and starvation+refeeding. The mRNA expression (n=5-6) was measured using qRT-PCR and the expression was normalized against five house keeping genes. The control for each group was set to 100 %. Graphpad Prism verison 5 was used to calcualte differences in mRNA expression using Mann-whitney (*>0.05, **>0.01, ***>0.001). Graphs display gene regulation of (AB) *Mfsd1*, (CD) *Slc59a1*, (EF) *Slc59a2*, (GH) *Slc33a2*, (IJ) *Slc60a1*, (KL) *Slc60a2*, (MN) *Slc61a1*, (OP) *Mfsd6*, (QR) *Mfsd8*, (ST) *Mfsd9*, (UV) *Slc22a32*, (WX) *Mfsd11*, (YZ) *Mfsd13a* and (AAAB) *Mfsd14a*.

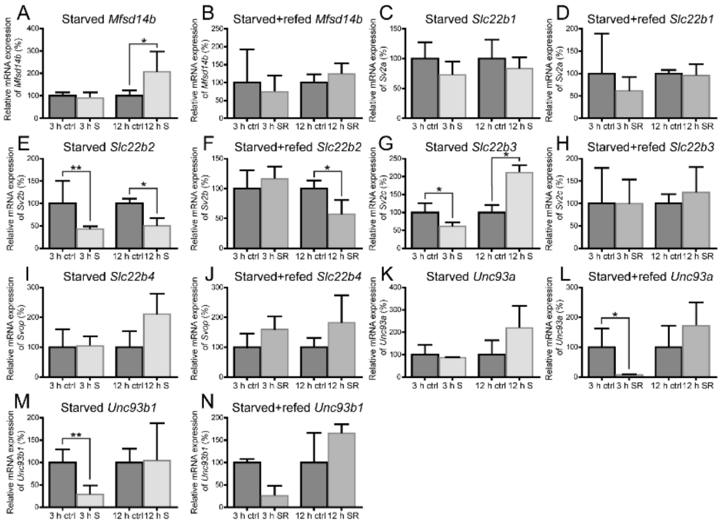

Supplementary figure 4: Gene regulation of putative SLCs in primary cortex cells subjected to 3 h and 12 h glucose starvation and starvation+refeeding. The mRNA expression (n=5-6) was measured using qRT-PCR and the expression was normalized against five house keeping genes. The control for each group was set to 100 %. Graphpad Prism verison 5 was used to calcual te differences in mRNA expression using Mann-whitney (*>0.05, **>0.01, ***>0.001). Graphs display gene regulation of (AB) *Mfsd14b*, (CD) *Slc22b1*, (EF) *Slc22b2*, (GH) *Slc22b3*, (IJ) *Slc22b4*, (KL) *Unc93a* and (MN) *Unc93b1*.

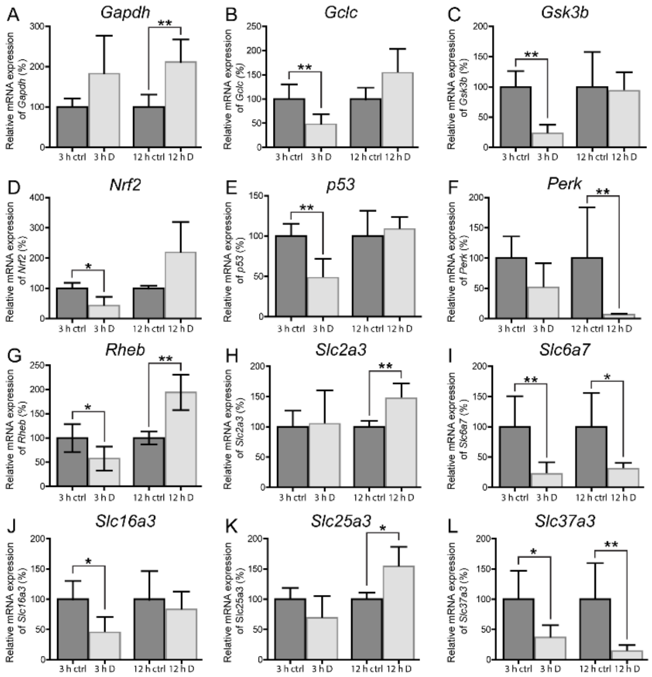

Supplementary figure 5: Relative mRNA expression for the metabolic and stress targets in glucose derpived primary cortex cultures. The mRNA expression (n=6) was measured using qRT-PCR and the expression was normalized against five house keeping genes. The control for each group was set to 100 %.

Graphpad Prism verison 5 was used to calcualte differences in mRNA expression using Mann-whitney (*>0.05, **>0.01, ***>0.001). Graphs display gene regulation of (A) *Gapdh*, (B) *Gclc*, (C) *Gsk3b*, (D) *Nrf2*, (E) *p53*, (F) *Perk*, (G) *Rheb*, (H) *Slc2a3*, (I) *Slc6a7*, (J) *Slc16a3*, (K) *Slc25a3* and (L) *Slc37a3*.

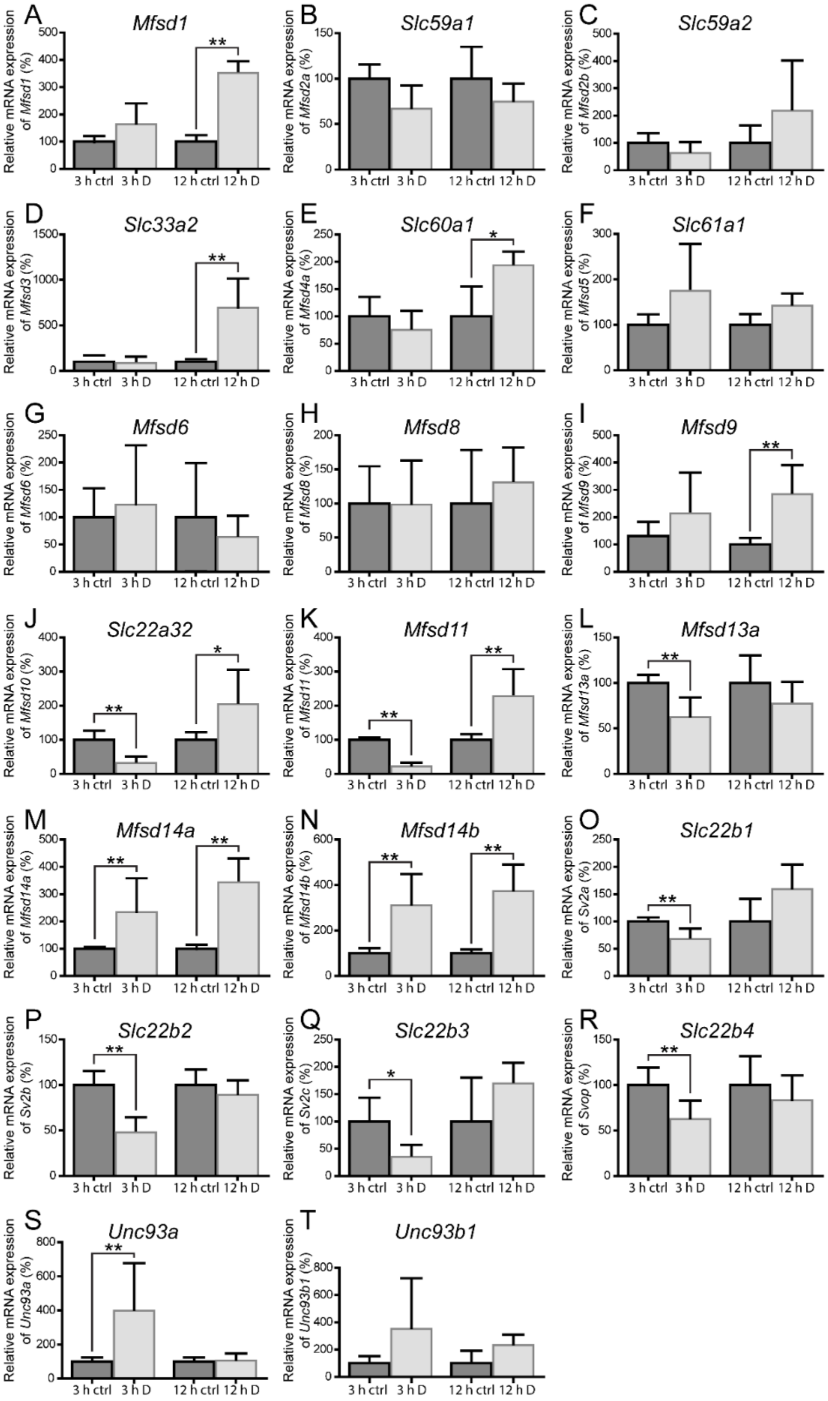

Supplementary figure 6: Gene regulation of putative SLCs in primary cortex cells subjected to 3 h and 12 h glucose derpivation. The mRNA expression (n=6) was measured using qRT-PCR and the expression was normalized against five house keeping genes. The control for each group was set to 100 %. Graphpad Prism verison 5 was used to calcualte differences in mRNA expression using Mann-whitney (*>0.05, **>0.01, ***>0.001). Graphs display gene regulation of (A) *Mfsd1*, (B) *Slc59a1*, (C) *Slc59a2*, (D) *Slc33a2*, (E)

Slc60a1, (F) *Slc61a1*, (G) *Mfsd6*, (H) *Mfsd8*, (I) *Mfsd9*, (J) *Slc22a32*, (K) *Mfsd11*, (L) *Mfsd13a*, (M) *Mfsd14a*, (N) *Mfsd14b*, (O) *Slc22b1*, (P) *Slc22b2*, (Q) *Slc22b3*, (R) *Slc22b4*, (S) *Unc93a* and (T) *Unc93b1*.

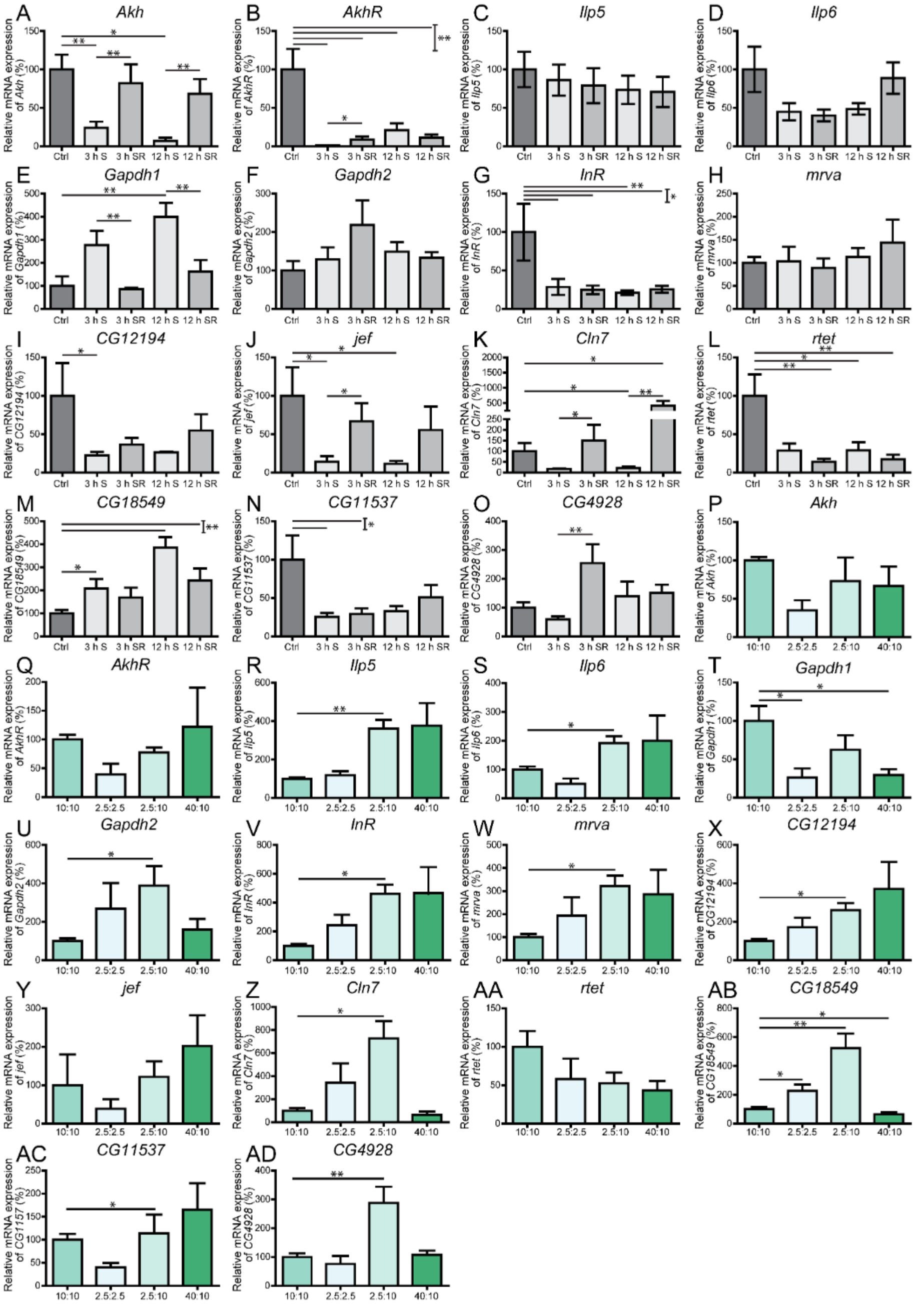

Supplementary figure 7: Relative mRNA expression for genes involved in metabolism and putative SLCs in fruit flies subjected to starvation, starvation and refeeding, low calorie diet, low sugar diet and high sugar diet. Flies were subjected to 0 h (n=6), 3 h starvation (n=8), 3 h starvation + refeeding (n=8), 12 h starvation (n=6) and 12 h starvation + refeeding (n=)6. Furthermore, adult male flies were maintained on diets containing different ratios of sugar and yeast for five days; normal diet (control 10:10 g), low calorie diet (2.5:2.5 g), low sugar (2.5:10 g) and high sugar (40:10 g). The gene expressions were measured using qRT-

PCR and the expression was normalized against three house keeping genes. Graphs were generated using Graphpad Prism version 5, were the control was set to 100 %, and differences were calculated using Kruskal-Wallis with Mann-Whitney as a posthoc test with Bonferroni's correction (*p<0.0489, **p<0.00995, ***p<0.00099). The relative mRNA expression after starvation as well as starvation and refeeding of (A) *Akh*, (B) *AkhR*, (C) *Ilp5*, (D) *Ilp6*, (E) *Gapdh1*, (F) *Gapdh2*, (G) *InR*, (H) *mrva*, (I) *CG12194*, (J) *jef*, (K) *Cln7*, (L) *rtet*, (M) *CG18549*, (N) *CG11537* and (O) *CG4928*. The relative mRNA expression of metabolic targets and putative SLCs in flies subjected to different diets (P) *Akh*, (Q) *AkhR*, (R) *Ilp5*, (S) *Ilp6*, (T) *Gapdh1*, (U) *Gapdh2*, (V) *InR*, (W) *mrva*, (X) *CG12194*, (Y) *jef*, (Z) *Cln7*, (AA), *rtet*, (AB) *CG18549*, (AC) *CG11537* and (AD) *CG4928*.
