# Supplementary material for: Glucose Availability Alters Gene and Protein Expression of Several Newly Classified and Putative Solute Carriers in Mice Cortex Cell Culture and D. melanogaster
Source: Front Cell Dev Biol. 2020 Jul 7;8:579. doi: 10.3389/fcell.2020.00579 (PMC7358622; doi:10.3389/fcell.2020.00579)
Supplement: Supplementary file 3 [file Data_Sheet_3.pdf]

CellProfiler Pipeline: http://www.cellprofiler.org

Version:4

DateRevision:318

GitHash:

ModuleCount:17

HasImagePlaneDetails:False

Images:[module_num:1|svn_version:\'Unknown\'|variable_revision_number:2|show_window:True|notes:\x5B\'To begin creating your project, use the Images module to compile a list of files and/or folders that you want to analyze. You can also specify a set of rules to include only the desired files in your selected folders.\'\x5D|batch_state:array(\x5B\x5D, dtype=uint8)|enabled:True|wants_pause:False]

:\xff\xfe

Filter images?:\xff\xfeI\x00m\x00a\x00g\x00e\x00s\x00 \x00o\x00n\x00l\x00y\x00

Select the rule criteria:\xff\xfea\x00n\x00d\x00 \x00(\x00f\x00i\x00l\x00e\x00 \x00d\x00o\x00e\x00s\x00 \x00c\x00o\x00n\x00t\x00a\x00i\x00n\x00 \x00"\x00c\x00t\x00r\x00l\x00"\x00)\x00

Metadata:[module_num:2|svn_version:\'Unknown\'|variable_revision_number:5|show_window:True|notes:\x5B\'The Metadata module optionally allows you to extract information describing your images (i.e, metadata) which will be stored along with your measurements. This information can be contained in the file name and/or location, or in an external file.\'\x5D|batch_state:array(\x5B\x5D, dtype=uint8)|enabled:True|wants_pause:False]

Extract metadata?:\xff\xfeY\x00e\x00s\x00

Metadata data type:\xff\xfeT\x00e\x00x\x00t\x00

Metadata types:\xff\xfe{\x00}\x00

Extraction method count:\xff\xfe1\x00

Metadata extraction method:\xff\xfeE\x00x\x00t\x00r\x00a\x00c\x00t\x00 \x00f\x00r\x00o\x00m\x00 \x00f\x00i\x00l\x00e\x00/\x00f\x00o\x00l\x00d\x00e\x00r\x00 \x00n\x00a\x00m\x00e\x00s\x00

Metadata source:\xff\xfeF\x00i\x00l\x00e\x00 \x00n\x00a\x00m\x00e\x00

Regular expression to extract from file name:\xff\xfe_\x00(\x00?\x00P\x00<\x00s\x00a\x00m\x00p\x00l\x00e\x00>\x00\x5B\x00a\x00-\x00z\x00\x5D\x00{\x004\x00}\x00)\x00_\x00(\x00?\x00P\x00<\x00P\x00i\x00c\x00N\x00u\x00m\x00b\x00e\x00r\x00>\x00\x5B\x000\x00-

\x009\x00\x5D\x00)\x00_\x00(\x00?\x00P\x00<\x00C\x00h\x00a\x00n\x00n\x00e\x00l\x00>\x00\x5B\x00a\x00-\x00z\x00\x5D\x00{\x003\x00,\x004\x00}\x00)\x00

Regular expression to extract from folder name:\xff\xfe(\x00?\x00P\x00<\x00D\x00a\x00t\x00e\x00>\x00\x5B\x000\x00-\x009\x00\x5D\x00{\x004\x00}\x00_\x00\x5B\x000\x00-\x009\x00\x5D\x00{\x002\x00}\x00_\x00\x5B\x000\x00-\x009\x00\x5D\x00{\x002\x00}\x00)\x00$\x00

Extract metadata from:\xff\xfeA\x00l\x00l\x00 \x00i\x00m\x00a\x00g\x00e\x00s\x00

Select the filtering criteria:\xff\xfea\x00n\x00d\x00 \x00(\x00f\x00i\x00l\x00e\x00 \x00d\x00o\x00e\x00s\x00 \x00c\x00o\x00n\x00t\x00a\x00i\x00n\x00 \x00"\x00"\x00)\x00

Metadata file location:\xff\xfeE\x00l\x00s\x00e\x00w\x00h\x00e\x00r\x00e\x00.\x00.\x00.\x00\x7C\x00

Match file and image metadata:\xff\xfe\x5B\x00\x5D\x00

Use case insensitive matching?:\xff\xfeN\x00o\x00

Metadata file name:\xff\xfe

NamesAndTypes:[module_num:3|svn_version:\'Unknown\'|variable_revision_number:8|show_window:True|notes:\x5B\'The NamesAndTypes module allows you to assign a meaningful name to each image by which other modules will refer to it.\'\x5D|batch_state:array(\x5B\x5D, dtype=uint8)|enabled:True|wants_pause:False]

Assign a name to:\xff\xfeI\x00m\x00a\x00g\x00e\x00s\x00 \x00m\x00a\x00t\x00c\x00h\x00i\x00n\x00g\x00 \x00r\x00u\x00l\x00e\x00s\x00

Select the image type:\xff\xfeG\x00r\x00a\x00y\x00s\x00c\x00a\x00l\x00e\x00 \x00i\x00m\x00a\x00g\x00e\x00

Name to assign these images:\xff\xfeD\x00N\x00A\x00

Match metadata:\xff\xfe\x5B\x00\x5D\x00

Image set matching method:\xff\xfeO\x00r\x00d\x00e\x00r\x00

Set intensity range from:\xff\xfeI\x00m\x00a\x00g\x00e\x00 \x00m\x00e\x00t\x00a\x00d\x00a\x00t\x00a\x00

Assignments count:\xff\xfe3\x00

Single images count:\xff\xfe0\x00

Maximum intensity:\xff\xfe2\x005\x005\x00.\x000\x00

Process as 3D?:\xff\xfeN\x00o\x00

Relative pixel spacing in X:\xff\xfe1\x00.\x000\x00

Relative pixel spacing in Y:\xff\xfe1\x00.\x000\x00

Relative pixel spacing in Z:\xff\xfe1\x00.\x000\x00

Select the rule criteria:\xff\xfeo\x00r\x00 \x00(\x00f\x00i\x00l\x00e\x00 \x00d\x00o\x00e\x00s\x00 \x00c\x00o\x00n\x00t\x00a\x00i\x00n\x00 \x00"\x00d\x00n\x00a\x00"\x00)\x00

Name to assign these images:\xff\xfeD\x00N\x00A\x00

Name to assign these objects:\xff\xfeC\x00e\x00l\x00l\x00

Select the image type:\xff\xfeC\x00o\x00l\x00o\x00r\x00 \x00i\x00m\x00a\x00g\x00e\x00

Set intensity range from:\xff\xfeI\x00m\x00a\x00g\x00e\x00 \x00m\x00e\x00t\x00a\x00d\x00a\x00t\x00a\x00

Maximum intensity:\xff\xfe2\x005\x005\x00.\x000\x00

Select the rule criteria:\xff\xfea\x00n\x00d\x00 \x00(\x00f\x00i\x00l\x00e\x00 \x00d\x00o\x00e\x00s\x00 \x00c\x00o\x00n\x00t\x00a\x00i\x00n\x00 \x00"\x00c\x00y\x00t\x00o\x00"\x00)\x00

Name to assign these images:\xff\xfec\x00y\x00t\x00o\x00

Name to assign these objects:\xff\xfeC\x00e\x00l\x00l\x00

Select the image type:\xff\xfeC\x00o\x00l\x00o\x00r\x00 \x00i\x00m\x00a\x00g\x00e\x00

Set intensity range from:\xff\xfeI\x00m\x00a\x00g\x00e\x00 \x00m\x00e\x00t\x00a\x00d\x00a\x00t\x00a\x00

Maximum intensity:\xff\xfe2\x005\x005\x00.\x000\x00

Select the rule criteria:\xff\xfea\x00n\x00d\x00 \x00(\x00f\x00i\x00l\x00e\x00 \x00d\x00o\x00e\x00s\x00 \x00c\x00o\x00n\x00t\x00a\x00i\x00n\x00 \x00"\x00F\x00I\x00T\x00C\x00"\x00)\x00

Name to assign these images:\xff\xfeg\x00f\x00p\x00

Name to assign these objects:\xff\xfeC\x00e\x00l\x00l\x00

Select the image type:\xff\xfeC\x00o\x00l\x00o\x00r\x00 \x00i\x00m\x00a\x00g\x00e\x00

Set intensity range from:\xff\xfeI\x00m\x00a\x00g\x00e\x00 \x00m\x00e\x00t\x00a\x00d\x00a\x00t\x00a\x00

Maximum intensity:\xff\xfe2\x005\x005\x00.\x000\x00

Groups:[module_num:4|svn_version:\'Unknown\'|variable_revision_number:2|show_window:True|notes:\x5B\'The Groups module optionally allows you to split your list of images into image subsets (groups) which will be processed independently of each other. Examples of groupings include screening

batches, microtiter plates, time-lapse movies, etc.\'\x5D|batch_state:array(\x5B\x5D, dtype=uint8)|enabled:True|wants_pause:False]

Do you want to group your images?:\xff\xfeY\x00e\x00s\x00

grouping metadata count:\xff\xfe1\x00

Metadata category:\xff\xfes\x00a\x00m\x00p\x00l\x00e\x00

ColorToGray:[module_num:5|svn_version:\'Unknown\'|variable_revision_number:4|show_window:True|notes:\x5B\x5D|batch_state:array(\x5B\x5D, dtype=uint8)|enabled:True|wants_pause:False]

Select the input image:\xff\xfeD\x00N\x00A\x00

Conversion method:\xff\xfeC\x00o\x00m\x00b\x00i\x00n\x00e\x00

Image type:\xff\xfeR\x00G\x00B\x00

Name the output image:\xff\xfeO\x00r\x00i\x00g\x00D\x00N\x00A\x00

Relative weight of the red channel:\xff\xfe0\x00

Relative weight of the green channel:\xff\xfe0\x00

Relative weight of the blue channel:\xff\xfe1\x00.\x000\x00

Convert red to gray?:\xff\xfeY\x00e\x00s\x00

Name the output image:\xff\xfeO\x00r\x00i\x00g\x00R\x00e\x00d\x00

Convert green to gray?:\xff\xfeY\x00e\x00s\x00

Name the output image:\xff\xfeO\x00r\x00i\x00g\x00G\x00r\x00e\x00e\x00n\x00

Convert blue to gray?:\xff\xfeY\x00e\x00s\x00

Name the output image:\xff\xfeO\x00r\x00i\x00g\x00B\x00l\x00u\x00e\x00

Convert hue to gray?:\xff\xfeY\x00e\x00s\x00

Name the output image:\xff\xfeO\x00r\x00i\x00g\x00H\x00u\x00e\x00

Convert saturation to gray?:\xff\xfeY\x00e\x00s\x00

Name the output image:\xff\xfeO\x00r\x00i\x00g\x00S\x00a\x00t\x00u\x00r\x00a\x00t\x00i\x00o\x00n\x00

Convert value to gray?:\xff\xfeY\x00e\x00s\x00

Name the output image:\xff\xfeO\x00r\x00i\x00g\x00V\x00a\x00l\x00u\x00e\x00

Channel count:\xff\xfe1\x00

Channel number:\xff\xfe1\x00

Relative weight of the channel:\xff\xfe1\x00.\x000\x00

Image name:\xff\xfeC\x00h\x00a\x00n\x00n\x00e\x00l\x001\x00

ColorToGray:[module_num:6|svn_version:\'Unknown\'|variable_revision_number:4|show_window:True|notes:\x5B\x5D|batch_state:array(\x5B\x5D, dtype=uint8)|enabled:True|wants_pause:False]

Select the input image:\xff\xfec\x00y\x00t\x00o\x00

Conversion method:\xff\xfeC\x00o\x00m\x00b\x00i\x00n\x00e\x00

Image type:\xff\xfeR\x00G\x00B\x00

Name the output image:\xff\xfeO\x00r\x00i\x00g\x00C\x00y\x00t\x00o\x00

Relative weight of the red channel:\xff\xfe1\x00

Relative weight of the green channel:\xff\xfe0\x00

Relative weight of the blue channel:\xff\xfe0\x00

Convert red to gray?:\xff\xfeY\x00e\x00s\x00

Name the output image:\xff\xfeO\x00r\x00i\x00g\x00R\x00e\x00d\x00

Convert green to gray?:\xff\xfeY\x00e\x00s\x00

Name the output image:\xff\xfeO\x00r\x00i\x00g\x00G\x00r\x00e\x00e\x00n\x00

Convert blue to gray?:\xff\xfeY\x00e\x00s\x00

Name the output image:\xff\xfeO\x00r\x00i\x00g\x00B\x00l\x00u\x00e\x00

Convert hue to gray?:\xff\xfeY\x00e\x00s\x00

Name the output image:\xff\xfeO\x00r\x00i\x00g\x00H\x00u\x00e\x00

Convert saturation to gray?:\xff\xfeY\x00e\x00s\x00

Name the output image:\xff\xfeO\x00r\x00i\x00g\x00S\x00a\x00t\x00u\x00r\x00a\x00t\x00i\x00o\x00n\x00

Convert value to gray?:\xff\xfeY\x00e\x00s\x00

Name the output image:\xff\xfeO\x00r\x00i\x00g\x00V\x00a\x00l\x00u\x00e\x00

Channel count:\xff\xfe1\x00

Channel number:\xff\xfe1\x00

Relative weight of the channel:\xff\xfe1\x00.\x000\x00

Image name:\xff\xfeC\x00h\x00a\x00n\x00n\x00e\x00l\x001\x00

ColorToGray:[module_num:7|svn_version:\'Unknown\'|variable_revision_number:4|show_window:True|notes:\x5B\x5D|batch_state:array(\x5B\x5D, dtype=uint8)|enabled:True|wants_pause:False]

Select the input image:\xff\xfeg\x00f\x00p\x00

Conversion method:\xff\xfeC\x00o\x00m\x00b\x00i\x00n\x00e\x00

Image type:\xff\xfeR\x00G\x00B\x00

Name the output image:\xff\xfeO\x00r\x00i\x00g\x00G\x00f\x00p\x00

Relative weight of the red channel:\xff\xfe0\x00

Relative weight of the green channel:\xff\xfe1\x00

Relative weight of the blue channel:\xff\xfe0\x00

Convert red to gray?:\xff\xfeY\x00e\x00s\x00

Name the output image:\xff\xfeO\x00r\x00i\x00g\x00R\x00e\x00d\x00

Convert green to gray?:\xff\xfeY\x00e\x00s\x00

Name the output image:\xff\xfeO\x00r\x00i\x00g\x00G\x00r\x00e\x00e\x00n\x00

Convert blue to gray?:\xff\xfeY\x00e\x00s\x00

Name the output image:\xff\xfeO\x00r\x00i\x00g\x00B\x00l\x00u\x00e\x00

Convert hue to gray?:\xff\xfeY\x00e\x00s\x00

Name the output image:\xff\xfeO\x00r\x00i\x00g\x00H\x00u\x00e\x00

Convert saturation to gray?:\xff\xfeY\x00e\x00s\x00

Name the output image:\xff\xfeO\x00r\x00i\x00g\x00S\x00a\x00t\x00u\x00r\x00a\x00t\x00i\x00o\x00n\x00

Convert value to gray?:\xff\xfeY\x00e\x00s\x00

Name the output image:\xff\xfeO\x00r\x00i\x00g\x00V\x00a\x00l\x00u\x00e\x00

Channel count:\xff\xfe1\x00

Channel number:\xff\xfe1\x00

Relative weight of the channel:\xff\xfe1\x00.\x000\x00

Image name:\xff\xfeC\x00h\x00a\x00n\x00n\x00e\x00l\x001\x00

IdentifyPrimaryObjects:[module_num:8|svn_version:\'Unknown\'|variable_revision_number:13|show_window:True|notes:\x5B\x5D|batch_state:array(\x5B\x5D, dtype=uint8)|enabled:True|wants_pause:False]

Select the input image:\xff\xfeO\x00r\x00i\x00g\x00D\x00N\x00A\x00

Name the primary objects to be identified:\xff\xfeN\x00u\x00c\x00l\x00e\x00i\x00

Typical diameter of objects, in pixel units (Min,Max):\xff\xfe3\x000\x00,\x002\x000\x000\x00

Discard objects outside the diameter range?:\xff\xfeY\x00e\x00s\x00

Discard objects touching the border of the image?:\xff\xfeY\x00e\x00s\x00

Method to distinguish clumped objects:\xff\xfeS\x00h\x00a\x00p\x00e\x00

Method to draw dividing lines between clumped objects:\xff\xfeI\x00n\x00t\x00e\x00n\x00s\x00i\x00t\x00y\x00

Size of smoothing filter:\xff\xfe1\x000\x00

Suppress local maxima that are closer than this minimum allowed distance:\xff\xfe7\x00.\x000\x00

Speed up by using lower-resolution image to find local maxima?:\xff\xfeY\x00e\x00s\x00

Fill holes in identified objects?:\xff\xfeA\x00f\x00t\x00e\x00r\x00 \x00b\x00o\x00t\x00h\x00 \x00t\x00h\x00r\x00e\x00s\x00h\x00o\x00l\x00d\x00i\x00n\x00g\x00 \x00a\x00n\x00d\x00 \x00d\x00e\x00c\x00l\x00u\x00m\x00p\x00i\x00n\x00g\x00

Automatically calculate size of smoothing filter for declumping?:\xff\xfeY\x00e\x00s\x00

Automatically calculate minimum allowed distance between local maxima?:\xff\xfeY\x00e\x00s\x00

Handling of objects if excessive number of objects identified:\xff\xfeC\x00o\x00n\x00t\x00i\x00n\x00u\x00e\x00

Maximum number of objects:\xff\xfe5\x000\x000\x00

Use advanced settings?:\xff\xfeY\x00e\x00s\x00

Threshold setting version:\xff\xfe9\x00

Threshold strategy:\xff\xfeG\x00l\x00o\x00b\x00a\x00l\x00

Thresholding method:\xff\xfeM\x00i\x00n\x00i\x00m\x00u\x00m\x00 \x00c\x00r\x00o\x00s\x00s\x00 \x00e\x00n\x00t\x00r\x00o\x00p\x00y\x00

Threshold smoothing scale:\xff\xfe1\x00.\x003\x004\x008\x008\x00

Threshold correction factor:\xff\xfe1\x00

Lower and upper bounds on threshold:\xff\xfe0\x00,\x00 \x001\x00

Manual threshold:\xff\xfe0\x00.\x001\x00

Select the measurement to threshold with:\xff\xfeN\x00o\x00n\x00e\x00

Two-class or three-class thresholding?:\xff\xfeT\x00w\x00o\x00 \x00c\x00l\x00a\x00s\x00s\x00e\x00s\x00

Assign pixels in the middle intensity class to the foreground or the background?:\xff\xfeF\x00o\x00r\x00e\x00g\x00r\x00o\x00u\x00n\x00d\x00

Size of adaptive window:\xff\xfe1\x000\x00

Lower outlier fraction:\xff\xfe0\x00.\x000\x005\x00

Upper outlier fraction:\xff\xfe0\x00.\x000\x005\x00

Averaging method:\xff\xfeM\x00e\x00a\x00n\x00

Variance method:\xff\xfeS\x00t\x00a\x00n\x00d\x00a\x00r\x00d\x00 \x00d\x00e\x00v\x00i\x00a\x00t\x00i\x00o\x00n\x00

# of deviations:\xff\xfe2\x00

Thresholding method:\xff\xfeO\x00t\x00s\x00u\x00

IdentifySecondaryObjects:[module_num:9|svn_version:\'Unknown\'|variable_revision_number:10|show_window:True|notes:\x5B\x5D|batch_state:array(\x5B\x5D, dtype=uint8)|enabled:True|wants_pause:False]

Select the input objects:\xff\xfeN\x00u\x00c\x00l\x00e\x00i\x00

Name the objects to be identified:\xff\xfeW\x00h\x00o\x00l\x00e\x00C\x00e\x00l\x00l\x00s\x00

Select the method to identify the secondary objects:\xff\xfeW\x00a\x00t\x00e\x00r\x00s\x00h\x00e\x00d\x00 \x00-\x00 \x00G\x00r\x00a\x00d\x00i\x00e\x00n\x00t\x00

Select the input image:\xff\xfeO\x00r\x00i\x00g\x00C\x00y\x00t\x00o\x00

Number of pixels by which to expand the primary objects:\xff\xfe1\x000\x00

Regularization factor:\xff\xfe0\x00.\x000\x005\x00

Discard secondary objects touching the border of the image?:\xff\xfeN\x00o\x00

Discard the associated primary objects?:\xff\xfeN\x00o\x00

Name the new primary objects:\xff\xfeF\x00i\x00l\x00t\x00e\x00r\x00e\x00d\x00N\x00u\x00c\x00l\x00e\x00i\x00

Fill holes in identified objects?:\xff\xfeN\x00o\x00

Threshold setting version:\xff\xfe9\x00

Threshold strategy:\xff\xfeG\x00l\x00o\x00b\x00a\x00l\x00

Thresholding method:\xff\xfeO\x00t\x00s\x00u\x00

Threshold smoothing scale:\xff\xfe0\x00

Threshold correction factor:\xff\xfe1\x00.\x000\x00

Lower and upper bounds on threshold:\xff\xfe0\x00.\x000\x00,\x001\x00.\x000\x00

Manual threshold:\xff\xfe0\x00.\x000\x00

Select the measurement to threshold with:\xff\xfeN\x00o\x00n\x00e\x00

Two-class or three-class thresholding?:\xff\xfeT\x00w\x00o\x00 \x00c\x00l\x00a\x00s\x00s\x00e\x00s\x00

Assign pixels in the middle intensity class to the foreground or the background?:\xff\xfeF\x00o\x00r\x00e\x00g\x00r\x00o\x00u\x00n\x00d\x00

Size of adaptive window:\xff\xfe1\x000\x00

Lower outlier fraction:\xff\xfe0\x00.\x000\x005\x00

Upper outlier fraction:\xff\xfe0\x00.\x000\x005\x00

Averaging method:\xff\xfeM\x00e\x00a\x00n\x00

Variance method:\xff\xfeS\x00t\x00a\x00n\x00d\x00a\x00r\x00d\x00 \x00d\x00e\x00v\x00i\x00a\x00t\x00i\x00o\x00n\x00

# of deviations:\xff\xfe2\x00

Thresholding method:\xff\xfeO\x00t\x00s\x00u\x00

IdentifySecondaryObjects:[module_num:10|svn_version:\'Unknown\'|variable_revision_number:10|show_window:True|notes:\x5B\x5D|batch_state:array(\x5B\x5D, dtype=uint8)|enabled:True|wants_pause:False]

Select the input objects:\xff\xfeN\x00u\x00c\x00l\x00e\x00i\x00

Name the objects to be identified:\xff\xfeC\x00o\x00r\x00e\x00C\x00e\x00l\x00l\x00s\x00

Select the method to identify the secondary objects:\xff\xfeW\x00a\x00t\x00e\x00r\x00s\x00h\x00e\x00d\x00 \x00-\x00 \x00G\x00r\x00a\x00d\x00i\x00e\x00n\x00t\x00

Select the input image:\xff\xfeO\x00r\x00i\x00g\x00C\x00y\x00t\x00o\x00

Number of pixels by which to expand the primary objects:\xff\xfe1\x000\x00

Regularization factor:\xff\xfe0\x00.\x000\x005\x00

Discard secondary objects touching the border of the image?:\xff\xfeN\x00o\x00

Discard the associated primary objects?:\xff\xfeN\x00o\x00

Name the new primary objects:\xff\xfeF\x00i\x00l\x00t\x00e\x00r\x00e\x00d\x00N\x00u\x00c\x00l\x00e\x00i\x00

Fill holes in identified objects?:\xff\xfeN\x00o\x00

Threshold setting version:\xff\xfe9\x00

Threshold strategy:\xff\xfeG\x00l\x00o\x00b\x00a\x00l\x00

Thresholding method:\xff\xfeM\x00a\x00n\x00u\x00a\x00l\x00

Threshold smoothing scale:\xff\xfe0\x00

Threshold correction factor:\xff\xfe1\x00.\x000\x00

Lower and upper bounds on threshold:\xff\xfe0\x00.\x000\x00,\x001\x00.\x000\x00

Manual threshold:\xff\xfe0\x00.\x002\x000\x00

Select the measurement to threshold with:\xff\xfeN\x00o\x00n\x00e\x00

Two-class or three-class thresholding?:\xff\xfeT\x00w\x00o\x00 \x00c\x00l\x00a\x00s\x00s\x00e\x00s\x00

Assign pixels in the middle intensity class to the foreground or the background?:\xff\xfeF\x00o\x00r\x00e\x00g\x00r\x00o\x00u\x00n\x00d\x00

Size of adaptive window:\xff\xfe1\x000\x00

Lower outlier fraction:\xff\xfe0\x00.\x000\x005\x00

Upper outlier fraction:\xff\xfe0\x00.\x000\x005\x00

Averaging method:\xff\xfeM\x00e\x00a\x00n\x00

Variance method:\xff\xfeS\x00t\x00a\x00n\x00d\x00a\x00r\x00d\x00 \x00d\x00e\x00v\x00i\x00a\x00t\x00i\x00o\x00n\x00

# of deviations:\xff\xfe2\x00

Thresholding method:\xff\xfeO\x00t\x00s\x00u\x00

IdentifyTertiaryObjects:[module_num:11|svn_version:\'Unknown\'|variable_revision_number:3|show_window:True|notes:\x5B\x5D|batch_state:array(\x5B\x5D, dtype=uint8)|enabled:True|wants_pause:False]

Select the larger identified objects:\xff\xfeW\x00h\x00o\x00l\x00e\x00C\x00e\x00l\x00l\x00s\x00

Select the smaller identified objects:\xff\xfeN\x00u\x00c\x00l\x00e\x00i\x00

Name the tertiary objects to be identified:\xff\xfeW\x00h\x00o\x00l\x00e\x00C\x00y\x00t\x00o\x00p\x00l\x00a\x00s\x00m\x00

Shrink smaller object prior to subtraction?:\xff\xfeY\x00e\x00s\x00

IdentifyTertiaryObjects:[module_num:12|svn_version:\'Unknown\'|variable_revision_number:3|show_window:True|notes:\x5B\x5D|batch_state:array(\x5B\x5D, dtype=uint8)|enabled:True|wants_pause:False]

Select the larger identified objects:\xff\xfeC\x00o\x00r\x00e\x00C\x00e\x00l\x00l\x00s\x00

Select the smaller identified objects:\xff\xfeN\x00u\x00c\x00l\x00e\x00i\x00

Name the tertiary objects to be identified:\xff\xfeC\x00o\x00r\x00e\x00C\x00y\x00t\x00o\x00p\x00l\x00a\x00s\x00m\x00

Shrink smaller object prior to subtraction?:\xff\xfeY\x00e\x00s\x00

IdentifyTertiaryObjects:[module_num:13|svn_version:\'Unknown\'|variable_revision_number:3|show_window:True|notes:\x5B\x5D|batch_state:array(\x5B\x5D, dtype=uint8)|enabled:True|wants_pause:False]

Select the larger identified objects:\xff\xfeW\x00h\x00o\x00l\x00e\x00C\x00e\x00l\x00l\x00s\x00

Select the smaller identified objects:\xff\xfeC\x00o\x00r\x00e\x00C\x00e\x00l\x00l\x00s\x00

Name the tertiary objects to be identified:\xff\xfeO\x00u\x00t\x00e\x00r\x00C\x00y\x00t\x00o\x00p\x00l\x00a\x00s\x00m\x00

Shrink smaller object prior to subtraction?:\xff\xfeY\x00e\x00s\x00

MeasureObjectIntensity:[module_num:14|svn_version:\'Unknown\'|variable_revision_number:3|show_window:True|notes:\x5B\x5D|batch_state:array(\x5B\x5D, dtype=uint8)|enabled:True|wants_pause:False]

Hidden:\xff\xfe1\x00

Select an image to measure:\xff\xfeO\x00r\x00i\x00g\x00G\x00f\x00p\x00

Select objects to measure:\xff\xfeW\x00h\x00o\x00l\x00e\x00C\x00y\x00t\x00o\x00p\x00l\x00a\x00s\x00m\x00

MeasureObjectIntensity:[module_num:15|svn_version:\'Unknown\'|variable_revision_number:3|show_window:True|notes:\x5B\x5D|batch_state:array(\x5B\x5D, dtype=uint8)|enabled:True|wants_pause:False]

Hidden:\xff\xfe1\x00

Select an image to measure:\xff\xfeO\x00r\x00i\x00g\x00G\x00f\x00p\x00

Select objects to measure:\xff\xfeC\x00o\x00r\x00e\x00C\x00y\x00t\x00o\x00p\x00l\x00a\x00s\x00m\x00

MeasureObjectIntensity:[module_num:16|svn_version:\'Unknown\'|variable_revision_number:3|show_window:True|notes:\x5B\x5D|batch_state:array(\x5B\x5D, dtype=uint8)|enabled:True|wants_pause:False]

Hidden:\xff\xfe1\x00

Select an image to measure:\xff\xfeO\x00r\x00i\x00g\x00G\x00f\x00p\x00

Select objects to measure:\xff\xfeO\x00u\x00t\x00e\x00r\x00C\x00y\x00t\x00o\x00p\x00l\x00a\x00s\x00m\x00

ExportToSpreadsheet:[module_num:17|svn_version:\'Unknown\'|variable_revision_number:12|show_window:True|notes:\x5B\x5D|batch_state:array(\x5B\x5D, dtype=uint8)|enabled:True|wants_pause:False]

Select the column delimiter:\xff\xfeC\x00o\x00m\x00m\x00a\x00 \x00(\x00"\x00,\x00"\x00)\x00

Add image metadata columns to your object data file?:\xff\xfeN\x00o\x00

Select the measurements to export:\xff\xfeY\x00e\x00s\x00

Calculate the per-image mean values for object measurements?:\xff\xfeN\x00o\x00

Calculate the per-image median values for object measurements?:\xff\xfeN\x00o\x00

Calculate the per-image standard deviation values for object measurements?:\xff\xfeN\x00o\x00

Output file location:\xff\xfeD\x00e\x00f\x00a\x00u\x00l\x00t\x00 \x00O\x00u\x00t\x00p\x00u\x00t\x00 \x00F\x00o\x00l\x00d\x00e\x00r\x00\x7C\x00

Create a GenePattern GCT file?:\xff\xfeN\x00o\x00

Select source of sample row name:\xff\xfeM\x00e\x00t\x00a\x00d\x00a\x00t\x00a\x00

Select the image to use as the identifier:\xff\xfeN\x00o\x00n\x00e\x00

Select the metadata to use as the identifier:\xff\xfeN\x00o\x00n\x00e\x00

Export all measurement types?:\xff\xfeY\x00e\x00s\x00

Press button to select measurements:\xff\xfeO\x00u\x00t\x00e\x00r\x00C\x00y\x00t\x00o\x00p\x00l\x00a\x00s\x00m\x00\x7C\x00I\x00n\x00t\x00e\x00n\x00s\x00i\x00t\x00y\x00_\x00M\x00a\x00s\x00s\x00D\x00i\x00s\x00p\

x00l\x00a\x00c\x00e\x00m\x00e\x00n\x00t\x00_\x00O\x00r\x00i\x00g\x00G\x00f\x00p\x00,\x00O\x0
0u\x00t\x00e\x00r\x00C\x00y\x00t\x00o\x00p\x00l\x00a\x00s\x00m\x00\x7C\x00I\x00n\x00t\x00e\x0
0n\x00s\x00i\x00t\x00y\x00_\x00M\x00i\x00n\x00I\x00n\x00t\x00e\x00n\x00s\x00i\x00t\x00y\x00_\x
00O\x00r\x00i\x00g\x00G\x00f\x00p\x00,\x00O\x00u\x00t\x00e\x00r\x00C\x00y\x00t\x00o\x00p\x00l
\x00a\x00s\x00m\x00\x7C\x00I\x00n\x00t\x00e\x00n\x00s\x00i\x00t\x00y\x00_\x00I\x00n\x00t\x00e
\x00g\x00r\x00a\x00t\x00e\x00d\x00I\x00n\x00t\x00e\x00n\x00s\x00i\x00t\x00y\x00E\x00d\x00g\x00
e\x00_\x00O\x00r\x00i\x00g\x00G\x00f\x00p\x00,\x00O\x00u\x00t\x00e\x00r\x00C\x00y\x00t\x00o\x
00p\x00l\x00a\x00s\x00m\x00\x7C\x00I\x00n\x00t\x00e\x00n\x00s\x00i\x00t\x00y\x00_\x00S\x00t\x
00d\x00I\x00n\x00t\x00e\x00n\x00s\x00i\x00t\x00y\x00_\x00O\x00r\x00i\x00g\x00G\x00f\x00p\x00,\
x00O\x00u\x00t\x00e\x00r\x00C\x00y\x00t\x00o\x00p\x00l\x00a\x00s\x00m\x00\x7C\x00I\x00n\x00t
\x00e\x00n\x00s\x00i\x00t\x00y\x00_\x00U\x00p\x00p\x00e\x00r\x00Q\x00u\x00a\x00r\x00t\x00i\x0
0l\x00e\x00I\x00n\x00t\x00e\x00n\x00s\x00i\x00t\x00y\x00_\x00O\x00r\x00i\x00g\x00G\x00f\x00p\x
00,\x00O\x00u\x00t\x00e\x00r\x00C\x00y\x00t\x00o\x00p\x00l\x00a\x00s\x00m\x00\x7C\x00I\x00n\x
00t\x00e\x00n\x00s\x00i\x00t\x00y\x00_\x00L\x00o\x00w\x00e\x00r\x00Q\x00u\x00a\x00r\x00t\x00i
\x00l\x00e\x00I\x00n\x00t\x00e\x00n\x00s\x00i\x00t\x00y\x00_\x00O\x00r\x00i\x00g\x00G\x00f\x00
p\x00,\x00O\x00u\x00t\x00e\x00r\x00C\x00y\x00t\x00o\x00p\x00l\x00a\x00s\x00m\x00\x7C\x00I\x00
n\x00t\x00e\x00n\x00s\x00i\x00t\x00y\x00_\x00M\x00i\x00n\x00I\x00n\x00t\x00e\x00n\x00s\x00i\x0
0t\x00y\x00E\x00d\x00g\x00e\x00_\x00O\x00r\x00i\x00g\x00G\x00f\x00p\x00,\x00O\x00u\x00t\x00e
\x00r\x00C\x00y\x00t\x00o\x00p\x00l\x00a\x00s\x00m\x00\x7C\x00I\x00n\x00t\x00e\x00n\x00s\x00i
\x00t\x00y\x00_\x00M\x00A\x00D\x00I\x00n\x00t\x00e\x00n\x00s\x00i\x00t\x00y\x00_\x00O\x00r\x
00i\x00g\x00G\x00f\x00p\x00,\x00O\x00u\x00t\x00e\x00r\x00C\x00y\x00t\x00o\x00p\x00l\x00a\x00s
\x00m\x00\x7C\x00I\x00n\x00t\x00e\x00n\x00s\x00i\x00t\x00y\x00_\x00M\x00e\x00a\x00n\x00I\x00
n\x00t\x00e\x00n\x00s\x00i\x00t\x00y\x00_\x00O\x00r\x00i\x00g\x00G\x00f\x00p\x00,\x00O\x00u\x
00t\x00e\x00r\x00C\x00y\x00t\x00o\x00p\x00l\x00a\x00s\x00m\x00\x7C\x00I\x00n\x00t\x00e\x00n\x
00s\x00i\x00t\x00y\x00_\x00I\x00n\x00t\x00e\x00g\x00r\x00a\x00t\x00e\x00d\x00I\x00n\x00t\x00e\
x00n\x00s\x00i\x00t\x00y\x00_\x00O\x00r\x00i\x00g\x00G\x00f\x00p\x00,\x00O\x00u\x00t\x00e\x00
r\x00C\x00y\x00t\x00o\x00p\x00l\x00a\x00s\x00m\x00\x7C\x00I\x00n\x00t\x00e\x00n\x00s\x00i\x00
t\x00y\x00_\x00M\x00e\x00a\x00n\x00I\x00n\x00t\x00e\x00n\x00s\x00i\x00t\x00y\x00E\x00d\x00g\x
00e\x00_\x00O\x00r\x00i\x00g\x00G\x00f\x00p\x00,\x00O\x00u\x00t\x00e\x00r\x00C\x00y\x00t\x00
o\x00p\x00l\x00a\x00s\x00m\x00\x7C\x00I\x00n\x00t\x00e\x00n\x00s\x00i\x00t\x00y\x00_\x00M\x0
0a\x00x\x00I\x00n\x00t\x00e\x00n\x00s\x00i\x00t\x00y\x00_\x00O\x00r\x00i\x00g\x00G\x00f\x00p\x
00,\x00O\x00u\x00t\x00e\x00r\x00C\x00y\x00t\x00o\x00p\x00l\x00a\x00s\x00m\x00\x7C\x00I\x00n\x
00t\x00e\x00n\x00s\x00i\x00t\x00y\x00_\x00M\x00e\x00d\x00i\x00a\x00n\x00I\x00n\x00t\x00e\x00n
\x00s\x00i\x00t\x00y\x00_\x00O\x00r\x00i\x00g\x00G\x00f\x00p\x00,\x00O\x00u\x00t\x00e\x00r\x00
C\x00y\x00t\x00o\x00p\x00l\x00a\x00s\x00m\x00\x7C\x00I\x00n\x00t\x00e\x00n\x00s\x00i\x00t\x00
y\x00_\x00S\x00t\x00d\x00I\x00n\x00t\x00e\x00n\x00s\x00i\x00t\x00y\x00E\x00d\x00g\x00e\x00_\x
00O\x00r\x00i\x00g\x00G\x00f\x00p\x00,\x00O\x00u\x00t\x00e\x00r\x00C\x00y\x00t\x00o\x00p\x00l
\x00a\x00s\x00m\x00\x7C\x00I\x00n\x00t\x00e\x00n\x00s\x00i\x00t\x00y\x00_\x00M\x00a\x00x\x00
I\x00n\x00t\x00e\x00n\x00s\x00i\x00t\x00y\x00E\x00d\x00g\x00e\x00_\x00O\x00r\x00i\x00g\x00G\x
00f\x00p\x00,\x00I\x00m\x00a\x00g\x00e\x00\x7C\x00F\x00i\x00l\x00e\x00N\x00a\x00m\x00e\x00_\
x00D\x00N\x00A\x00,\x00I\x00m\x00a\x00g\x00e\x00\x7C\x00M\x00e\x00t\x00a\x00d\x00a\x00t\x0
0a\x00_\x00s\x00a\x00m\x00p\x00l\x00e\x00,\x00I\x00m\x00a\x00g\x00e\x00\x7C\x00M\x00e\x00t\
x00a\x00d\x00a\x00t\x00a\x00_\x00P\x00i\x00c\x00N\x00u\x00m\x00b\x00e\x00r\x00,\x00W\x00h\x
00o\x00l\x00e\x00C\x00y\x00t\x00o\x00p\x00l\x00a\x00s\x00m\x00\x7C\x00I\x00n\x00t\x00e\x00n\x

00s\x00i\x00t\x00y\x00_\x00M\x00a\x00s\x00s\x00D\x00i\x00s\x00p\x00l\x00a\x00c\x00e\x00m\x00
e\x00n\x00t\x00_\x00O\x00r\x00i\x00g\x00G\x00f\x00p\x00,\x00W\x00h\x00o\x00l\x00e\x00C\x00y\
x00t\x00o\x00p\x00l\x00a\x00s\x00m\x00\x7C\x00I\x00n\x00t\x00e\x00n\x00s\x00i\x00t\x00y\x00_\
x00M\x00i\x00n\x00I\x00n\x00t\x00e\x00n\x00s\x00i\x00t\x00y\x00_\x00O\x00r\x00i\x00g\x00G\x00
f\x00p\x00,\x00W\x00h\x00o\x00l\x00e\x00C\x00y\x00t\x00o\x00p\x00l\x00a\x00s\x00m\x00\x7C\x0
0I\x00n\x00t\x00e\x00n\x00s\x00i\x00t\x00y\x00_\x00I\x00n\x00t\x00e\x00g\x00r\x00a\x00t\x00e\x
00d\x00I\x00n\x00t\x00e\x00n\x00s\x00i\x00t\x00y\x00E\x00d\x00g\x00e\x00_\x00O\x00r\x00i\x00g
\x00G\x00f\x00p\x00,\x00W\x00h\x00o\x00l\x00e\x00C\x00y\x00t\x00o\x00p\x00l\x00a\x00s\x00m\x
00\x7C\x00I\x00n\x00t\x00e\x00n\x00s\x00i\x00t\x00y\x00_\x00S\x00t\x00d\x00I\x00n\x00t\x00e\x0
0n\x00s\x00i\x00t\x00y\x00_\x00O\x00r\x00i\x00g\x00G\x00f\x00p\x00,\x00W\x00h\x00o\x00l\x00e\
x00C\x00y\x00t\x00o\x00p\x00l\x00a\x00s\x00m\x00\x7C\x00I\x00n\x00t\x00e\x00n\x00s\x00i\x00t\
x00y\x00_\x00U\x00p\x00p\x00e\x00r\x00Q\x00u\x00a\x00r\x00t\x00i\x00l\x00e\x00I\x00n\x00t\x00
e\x00n\x00s\x00i\x00t\x00y\x00_\x00O\x00r\x00i\x00g\x00G\x00f\x00p\x00,\x00W\x00h\x00o\x00l\x
00e\x00C\x00y\x00t\x00o\x00p\x00l\x00a\x00s\x00m\x00\x7C\x00I\x00n\x00t\x00e\x00n\x00s\x00i\x
00t\x00y\x00_\x00I\x00n\x00t\x00e\x00g\x00r\x00a\x00t\x00e\x00d\x00I\x00n\x00t\x00e\x00n\x00s\
x00i\x00t\x00y\x00_\x00O\x00r\x00i\x00g\x00G\x00f\x00p\x00,\x00W\x00h\x00o\x00l\x00e\x00C\x0
0y\x00t\x00o\x00p\x00l\x00a\x00s\x00m\x00\x7C\x00I\x00n\x00t\x00e\x00n\x00s\x00i\x00t\x00y\x0
0_\x00M\x00i\x00n\x00I\x00n\x00t\x00e\x00n\x00s\x00i\x00t\x00y\x00E\x00d\x00g\x00e\x00_\x00O
\x00r\x00i\x00g\x00G\x00f\x00p\x00,\x00W\x00h\x00o\x00l\x00e\x00C\x00y\x00t\x00o\x00p\x00l\x0
0a\x00s\x00m\x00\x7C\x00I\x00n\x00t\x00e\x00n\x00s\x00i\x00t\x00y\x00_\x00M\x00A\x00D\x00I\x
00n\x00t\x00e\x00n\x00s\x00i\x00t\x00y\x00_\x00O\x00r\x00i\x00g\x00G\x00f\x00p\x00,\x00W\x00
h\x00o\x00l\x00e\x00C\x00y\x00t\x00o\x00p\x00l\x00a\x00s\x00m\x00\x7C\x00I\x00n\x00t\x00e\x00
n\x00s\x00i\x00t\x00y\x00_\x00M\x00e\x00a\x00n\x00I\x00n\x00t\x00e\x00n\x00s\x00i\x00t\x00y\x
00_\x00O\x00r\x00i\x00g\x00G\x00f\x00p\x00,\x00W\x00h\x00o\x00l\x00e\x00C\x00y\x00t\x00o\x00
p\x00l\x00a\x00s\x00m\x00\x7C\x00I\x00n\x00t\x00e\x00n\x00s\x00i\x00t\x00y\x00_\x00M\x00e\x0
0a\x00n\x00I\x00n\x00t\x00e\x00n\x00s\x00i\x00t\x00y\x00E\x00d\x00g\x00e\x00_\x00O\x00r\x00i\
x00g\x00G\x00f\x00p\x00,\x00W\x00h\x00o\x00l\x00e\x00C\x00y\x00t\x00o\x00p\x00l\x00a\x00s\x0
0m\x00\x7C\x00I\x00n\x00t\x00e\x00n\x00s\x00i\x00t\x00y\x00_\x00L\x00o\x00w\x00e\x00r\x00Q\x
00u\x00a\x00r\x00t\x00i\x00l\x00e\x00I\x00n\x00t\x00e\x00n\x00s\x00i\x00t\x00y\x00_\x00O\x00r\x
00i\x00g\x00G\x00f\x00p\x00,\x00W\x00h\x00o\x00l\x00e\x00C\x00y\x00t\x00o\x00p\x00l\x00a\x00
s\x00m\x00\x7C\x00I\x00n\x00t\x00e\x00n\x00s\x00i\x00t\x00y\x00_\x00M\x00a\x00x\x00I\x00n\x0
0t\x00e\x00n\x00s\x00i\x00t\x00y\x00_\x00O\x00r\x00i\x00g\x00G\x00f\x00p\x00,\x00W\x00h\x00o\
x00l\x00e\x00C\x00y\x00t\x00o\x00p\x00l\x00a\x00s\x00m\x00\x7C\x00I\x00n\x00t\x00e\x00n\x00s\
x00i\x00t\x00y\x00_\x00M\x00e\x00d\x00i\x00a\x00n\x00I\x00n\x00t\x00e\x00n\x00s\x00i\x00t\x00
y\x00_\x00O\x00r\x00i\x00g\x00G\x00f\x00p\x00,\x00W\x00h\x00o\x00l\x00e\x00C\x00y\x00t\x00o\
x00p\x00l\x00a\x00s\x00m\x00\x7C\x00I\x00n\x00t\x00e\x00n\x00s\x00i\x00t\x00y\x00_\x00S\x00t\
x00d\x00I\x00n\x00t\x00e\x00n\x00s\x00i\x00t\x00y\x00E\x00d\x00g\x00e\x00_\x00O\x00r\x00i\x00
g\x00G\x00f\x00p\x00,\x00W\x00h\x00o\x00l\x00e\x00C\x00y\x00t\x00o\x00p\x00l\x00a\x00s\x00m\
x00\x7C\x00I\x00n\x00t\x00e\x00n\x00s\x00i\x00t\x00y\x00_\x00M\x00a\x00x\x00I\x00n\x00t\x00e\
x00n\x00s\x00i\x00t\x00y\x00E\x00d\x00g\x00e\x00_\x00O\x00r\x00i\x00g\x00G\x00f\x00p\x00,\x00
C\x00o\x00r\x00e\x00C\x00y\x00t\x00o\x00p\x00l\x00a\x00s\x00m\x00\x7C\x00I\x00n\x00t\x00e\x0
0n\x00s\x00i\x00t\x00y\x00_\x00M\x00a\x00s\x00s\x00D\x00i\x00s\x00p\x00l\x00a\x00c\x00e\x00m
\x00e\x00n\x00t\x00_\x00O\x00r\x00i\x00g\x00G\x00f\x00p\x00,\x00C\x00o\x00r\x00e\x00C\x00y\x0
0t\x00o\x00p\x00l\x00a\x00s\x00m\x00\x7C\x00I\x00n\x00t\x00e\x00n\x00s\x00i\x00t\x00y\x00_\x0

0M\x00i\x00n\x00I\x00n\x00t\x00e\x00n\x00s\x00i\x00t\x00y\x00_\x00O\x00r\x00i\x00g\x00G\x00f\
x00p\x00,\x00C\x00o\x00r\x00e\x00C\x00y\x00t\x00o\x00p\x00l\x00a\x00s\x00m\x00\x7C\x00I\x00n
\x00t\x00e\x00n\x00s\x00i\x00t\x00y\x00_\x00S\x00t\x00d\x00I\x00n\x00t\x00e\x00n\x00s\x00i\x00t
\x00y\x00_\x00O\x00r\x00i\x00g\x00G\x00f\x00p\x00,\x00C\x00o\x00r\x00e\x00C\x00y\x00t\x00o\x0
0p\x00l\x00a\x00s\x00m\x00\x7C\x00I\x00n\x00t\x00e\x00n\x00s\x00i\x00t\x00y\x00_\x00I\x00n\x0
0t\x00e\x00g\x00r\x00a\x00t\x00e\x00d\x00I\x00n\x00t\x00e\x00n\x00s\x00i\x00t\x00y\x00E\x00d\x
00g\x00e\x00_\x00O\x00r\x00i\x00g\x00G\x00f\x00p\x00,\x00C\x00o\x00r\x00e\x00C\x00y\x00t\x00
o\x00p\x00l\x00a\x00s\x00m\x00\x7C\x00I\x00n\x00t\x00e\x00n\x00s\x00i\x00t\x00y\x00_\x00M\x0
0i\x00n\x00I\x00n\x00t\x00e\x00n\x00s\x00i\x00t\x00y\x00E\x00d\x00g\x00e\x00_\x00O\x00r\x00i\x
00g\x00G\x00f\x00p\x00,\x00C\x00o\x00r\x00e\x00C\x00y\x00t\x00o\x00p\x00l\x00a\x00s\x00m\x00
\x7C\x00I\x00n\x00t\x00e\x00n\x00s\x00i\x00t\x00y\x00_\x00L\x00o\x00w\x00e\x00r\x00Q\x00u\x0
0a\x00r\x00t\x00i\x00l\x00e\x00I\x00n\x00t\x00e\x00n\x00s\x00i\x00t\x00y\x00_\x00O\x00r\x00i\x0
0g\x00G\x00f\x00p\x00,\x00C\x00o\x00r\x00e\x00C\x00y\x00t\x00o\x00p\x00l\x00a\x00s\x00m\x00\
x7C\x00I\x00n\x00t\x00e\x00n\x00s\x00i\x00t\x00y\x00_\x00U\x00p\x00p\x00e\x00r\x00Q\x00u\x00
a\x00r\x00t\x00i\x00l\x00e\x00I\x00n\x00t\x00e\x00n\x00s\x00i\x00t\x00y\x00_\x00O\x00r\x00i\x00
g\x00G\x00f\x00p\x00,\x00C\x00o\x00r\x00e\x00C\x00y\x00t\x00o\x00p\x00l\x00a\x00s\x00m\x00\x
7C\x00I\x00n\x00t\x00e\x00n\x00s\x00i\x00t\x00y\x00_\x00M\x00A\x00D\x00I\x00n\x00t\x00e\x00n
\x00s\x00i\x00t\x00y\x00_\x00O\x00r\x00i\x00g\x00G\x00f\x00p\x00,\x00C\x00o\x00r\x00e\x00C\x0
0y\x00t\x00o\x00p\x00l\x00a\x00s\x00m\x00\x7C\x00I\x00n\x00t\x00e\x00n\x00s\x00i\x00t\x00y\x0
0_\x00M\x00e\x00a\x00n\x00I\x00n\x00t\x00e\x00n\x00s\x00i\x00t\x00y\x00_\x00O\x00r\x00i\x00g\
x00G\x00f\x00p\x00,\x00C\x00o\x00r\x00e\x00C\x00y\x00t\x00o\x00p\x00l\x00a\x00s\x00m\x00\x7C
\x00I\x00n\x00t\x00e\x00n\x00s\x00i\x00t\x00y\x00_\x00I\x00n\x00t\x00e\x00g\x00r\x00a\x00t\x00
e\x00d\x00I\x00n\x00t\x00e\x00n\x00s\x00i\x00t\x00y\x00_\x00O\x00r\x00i\x00g\x00G\x00f\x00p\x
00,\x00C\x00o\x00r\x00e\x00C\x00y\x00t\x00o\x00p\x00l\x00a\x00s\x00m\x00\x7C\x00I\x00n\x00t\x
00e\x00n\x00s\x00i\x00t\x00y\x00_\x00M\x00e\x00a\x00n\x00I\x00n\x00t\x00e\x00n\x00s\x00i\x00t
\x00y\x00E\x00d\x00g\x00e\x00_\x00O\x00r\x00i\x00g\x00G\x00f\x00p\x00,\x00C\x00o\x00r\x00e\x
00C\x00y\x00t\x00o\x00p\x00l\x00a\x00s\x00m\x00\x7C\x00I\x00n\x00t\x00e\x00n\x00s\x00i\x00t\x
00y\x00_\x00M\x00a\x00x\x00I\x00n\x00t\x00e\x00n\x00s\x00i\x00t\x00y\x00_\x00O\x00r\x00i\x00g
\x00G\x00f\x00p\x00,\x00C\x00o\x00r\x00e\x00C\x00y\x00t\x00o\x00p\x00l\x00a\x00s\x00m\x00\x7
C\x00I\x00n\x00t\x00e\x00n\x00s\x00i\x00t\x00y\x00_\x00M\x00e\x00d\x00i\x00a\x00n\x00I\x00n\x
00t\x00e\x00n\x00s\x00i\x00t\x00y\x00_\x00O\x00r\x00i\x00g\x00G\x00f\x00p\x00,\x00C\x00o\x00r\
x00e\x00C\x00y\x00t\x00o\x00p\x00l\x00a\x00s\x00m\x00\x7C\x00I\x00n\x00t\x00e\x00n\x00s\x00i\
x00t\x00y\x00_\x00S\x00t\x00d\x00I\x00n\x00t\x00e\x00n\x00s\x00i\x00t\x00y\x00E\x00d\x00g\x00
e\x00_\x00O\x00r\x00i\x00g\x00G\x00f\x00p\x00,\x00C\x00o\x00r\x00e\x00C\x00y\x00t\x00o\x00p\x
00l\x00a\x00s\x00m\x00\x7C\x00I\x00n\x00t\x00e\x00n\x00s\x00i\x00t\x00y\x00_\x00M\x00a\x00x\
x00I\x00n\x00t\x00e\x00n\x00s\x00i\x00t\x00y\x00E\x00d\x00g\x00e\x00_\x00O\x00r\x00i\x00g\x00
G\x00f\x00p\x00

Representation of Nan/Inf:\xff\xfeN\x00a\x00N\x00

Add a prefix to file names?:\xff\xfeY\x00e\x00s\x00

Filename prefix:\xff\xfeT\x00e\x00s\x00t\x002\x00_\x00

Overwrite existing files without warning?:\xff\xfeN\x00o\x00

Data to export:\xff\xfeD\x00o\x00 \x00n\x00o\x00t\x00 \x00u\x00s\x00e\x00

Combine these object measurements with those of the previous object?:\xff\xfeN\x00o\x00

File name:\xff\xfeD\x00A\x00T\x00A\x00.\x00c\x00s\x00v\x00

Use the object name for the file name?:\xff\xfeY\x00e\x00s\x00
